# Incorporation of genetic model parameters for cost-effective designs of genetic association studies using DNA pooling

**DOI:** 10.1186/1471-2164-8-238

**Published:** 2007-07-16

**Authors:** Fei Ji, Stephen J Finch, Chad Haynes, Nancy R Mendell, Derek Gordon

**Affiliations:** 1 Lab of Statistical Genetics, Rockefeller University, New York, NY, USA; 2 Department of Applied Math and Statistics, Stony Brook University, Stony Brook, NY, USA; 3 Department of Genetics, Rutgers University, Piscataway, NJ, USA

## Abstract

**Background:**

Studies of association methods using DNA pooling of single nucleotide polymorphisms (SNPs) have focused primarily on the effects of "machine-error", number of replicates, and the size of the pool. We use the non-centrality parameter (NCP) for the analysis of variance test to compute the approximate power for genetic association tests with DNA pooling data on cases and controls. We incorporate genetic model parameters into the computation of the NCP. Parameters involved in the power calculation are disease allele frequency, frequency of the marker SNP allele in coupling with the disease locus, disease prevalence, genotype relative risk, sample size, genetic model, number of pools, number of replicates of each pool, and the proportion of variance of the pooled frequency estimate due to machine variability. We compute power for different settings of number of replicates and total number of genotypings when the genetic model parameters are fixed. Several significance levels are considered, including stringent significance levels (due to the increasing popularity of 100 K and 500 K SNP "chip" data). We use a factorial design with two to four settings of each parameter and multiple regression analysis to assess which parameters most significantly affect power.

**Results:**

The power can increase substantially as the genotyping number increases. For a fixed number of genotypings, the power is a function of the number of replicates of each pool such that there is a setting with maximum power. The four most significant parameters affecting power for association are: (1) genotype relative risk, (2) genetic model, (3) sample size, and (4) the interaction term between disease and SNP marker allele probabilities.

**Conclusion:**

For a fixed number of genotypings, there is an optimal number of replicates of each pool that increases as the number of genotypings increases. Power is not substantially reduced when the number of replicates is close to but not equal to the optimal setting.

## Background

Case/control genetic association studies are used as a means of localizing susceptibility genes for a complex disease. With the recent development of technologies that can determine the genotypes for hundreds of thousands of single nucleotide polymorphisms (SNPs) across the human genome, such studies are now being reported in the literature [1-3]. Design issues such as power to detect association using these technologies are also being published [4,5]. Since a critical requirement for such studies to be sufficiently powered is that the disequilibrium among the disease allele and neighboring marker alleles be large, marker density needs to be high. If the effect size for a complex disease is small (e.g., genotype relative risks [6] on the order of 1.5 to 2), the sample size required to detect association may be thousands of cases and controls [4,5,7-9]. Therefore, researchers often consider genotyping technologies such as DNA pooling [10-13] as an initial strategy to identify genomic regions that may harbor susceptibility loci in an effort to reduce cost (time and money) (e.g.,[14,15]). Advantages of DNA pooling technologies include (a sometimes substantial) reduction in genotyping cost when performing multi-stage association studies to identify disease susceptibility genes. Potential disadvantages include reliance on a number of assumptions related to statistical design and analysis. For example, a key assumption is that the intensity measure has an expected value equal to the allele frequency. Another potential disadvantage is that DNA pooling techniques may not detect disease mode of inheritances that deviate from dominant or recessive modes. For example, DNA pooling techniques will be underpowered to detect disease genes that operate in an over-dominant form.

Sham et al. reviewed currently available technologies for DNA pooling [10]. The statistical analysis of data from pooled DNA studies uses analysis of variance (ANOVA) procedures that have algorithms for calculating power to detect unequal allele probabilities. A major design issue when using DNA pooling technologies is the measurement error as compared with the gold standard method of individual genotyping.

Research has been done regarding specification of study parameter settings to maximize power [10,16,17]. The research question addressed in this work is: assuming a certain level of measurement error, what settings of study design parameters maximize the power to detect association? More specifically, we study the sensitivity of power to changes in design parameters (e.g., total sample size, differing numbers of genotypings, number of pools, and genetic model parameters). We present a closed form approximation to the power in terms of the genetic model, pooling measurement error model, and the study parameters (e.g., number of pools, number of replicates per pool, sample size) and we perform a systematic study of the design parameters to identify which have the greatest effect on power to detect association for DNA pooling studies.

## Results

The pooled DNA association studies considered here have equal number of cases and controls *N*. For a fixed number of total subjects (cases and controls), an equal number of cases and controls yields maximal power for association [7,8]. The *N *subjects in each group are randomly assigned to one of *J *pools, each of size *T *(so that *N *= *J *× *T*). Each of the *J *pools has *K *replicate measures, so that the number of case genotypings is equal to the number of control genotypings (*G *= *J *× *K*). The data analyzed in the study are the estimated allele frequencies *Y*_*ijk *_of the more common allele (called "2"), where the index *i *is 0 for cases and 1 for controls, the index *j *ranges from 1 to *J*, and the index *k *ranges from 1 to *K*. The variance of *Y*_*ijk *_has two components, one due to the sampling variability of the frequency of allele 2 in each pool (denoted by σP,i2
 MathType@MTEF@5@5@+=feaafiart1ev1aaatCvAUfKttLearuWrP9MDH5MBPbIqV92AaeXatLxBI9gBaebbnrfifHhDYfgasaacH8akY=wiFfYdH8Gipec8Eeeu0xXdbba9frFj0=OqFfea0dXdd9vqai=hGuQ8kuc9pgc9s8qqaq=dirpe0xb9q8qiLsFr0=vr0=vr0dc8meaabaqaciaacaGaaeqabaqabeGadaaakeaaiiGacqWFdpWCdaqhaaWcbaGaemiuaaLaeiilaWIaemyAaKgabaGaeGOmaidaaaaa@32F9@ here) and the other due to the variability of the measurement process of the pooled material (denoted by σE2=σP,i2(m−1)
 MathType@MTEF@5@5@+=feaafiart1ev1aaatCvAUfKttLearuWrP9MDH5MBPbIqV92AaeXatLxBI9gBaebbnrfifHhDYfgasaacH8akY=wiFfYdH8Gipec8Eeeu0xXdbba9frFj0=OqFfea0dXdd9vqai=hGuQ8kuc9pgc9s8qqaq=dirpe0xb9q8qiLsFr0=vr0=vr0dc8meaabaqaciaacaGaaeqabaqabeGadaaakeaaiiGacqWFdpWCdaqhaaWcbaGaemyraueabaGaeGOmaidaaOGaeyypa0Jae83Wdm3aa0baaSqaaiabdcfaqjabcYcaSiabdMgaPbqaaiabikdaYaaakiabcIcaOiabd2gaTjabgkHiTiabigdaXiabcMcaPaaa@3CF5@ here). We refer to the term *m *as the measure of the *machine replicability variance factor*. The quality of the estimate of the pooled frequency as measured by its variance is parameterized so that is proportional to the sampling variance of the allele 2 frequency and is assumed to be independent of pool size or other pooling parameters. When the number of pools is *J *≥ 2, the structure of a pooled DNA study is an example of a two-stage nested design [18]. Its statistical analysis is conventionally organized in an ANOVA table as in Table 1, with the null hypothesis that the case allele 2 frequency is equal to the control allele 2 frequency. This hypothesis is tested using the statistic F=SSA/1SSP/[2(J−1)]=MSAMSP
 MathType@MTEF@5@5@+=feaafiart1ev1aaatCvAUfKttLearuWrP9MDH5MBPbIqV92AaeXatLxBI9gBaebbnrfifHhDYfgasaacH8akY=wiFfYdH8Gipec8Eeeu0xXdbba9frFj0=OqFfea0dXdd9vqai=hGuQ8kuc9pgc9s8qqaq=dirpe0xb9q8qiLsFr0=vr0=vr0dc8meaabaqaciaacaGaaeqabaqabeGadaaakeaacqWGgbGrcqGH9aqpdaWcaaqaaiabdofatjabdofatnaaBaaaleaacqWGbbqqaeqaaOGaei4la8IaeGymaedabaGaem4uamLaem4uam1aaSbaaSqaaiabdcfaqbqabaGccqGGVaWlcqGGBbWwcqaIYaGmcqGGOaakcqWGkbGscqGHsislcqaIXaqmcqGGPaqkcqGGDbqxaaGaeyypa0ZaaSaaaeaacqWGnbqtcqWGtbWudaWgaaWcbaGaemyqaeeabeaaaOqaaiabd2eanjabdofatnaaBaaaleaacqWGqbauaeqaaaaaaaa@495D@. Here, *SS*_*A *_is the sum of squares of the case/control averages, *SS*_*P *_is the sum of squares of the pool averages within a group about the group pool mean, and is the basis of an estimate of the variance of a pool average frequency. The term *MS*_*A *_is the mean square of *SS*_*A*_, which by definition is just *SS*_*A *_divided by the degrees of freedom (df), and similarly for *MS*_*P*_. Under the null hypothesis, *MS*_*A *_is also the basis of an estimate of the variance of a pool average frequency. When the null hypothesis is false, on average, *MS*_*A *_is increased as shown in its expected mean square.

**Table 1:** The analysis of variance table for a two-stage nested design

ANOVA Table			
Source	DF	SS	E(MS)
Case or control (*α*)	1	∑i∑j∑k(Yi••−Y•••)2=JK∑i(Yi••−Y•••)2 MathType@MTEF@5@5@+=feaafiart1ev1aaatCvAUfKttLearuWrP9MDH5MBPbIqV92AaeXatLxBI9gBaebbnrfifHhDYfgasaacH8akY=wiFfYdH8Gipec8Eeeu0xXdbba9frFj0=OqFfea0dXdd9vqai=hGuQ8kuc9pgc9s8qqaq=dirpe0xb9q8qiLsFr0=vr0=vr0dc8meaabaqaciaacaGaaeqabaqabeGadaaakeaadaaeqbqaamaaqafabaWaaabuaeaacqGGOaakcqWGzbqwdaWgaaWcbaGaemyAaKMaeyOiGCRaeyOiGClabeaakiabgkHiTaWcbaGaem4AaSgabeqdcqGHris5aaWcbaGaemOAaOgabeqdcqGHris5aaWcbaGaemyAaKgabeqdcqGHris5aOGaemywaK1aaSbaaSqaaiabgkci3kabgkci3kabgkci3cqabaGccqGGPaqkdaahaaWcbeqaaiabikdaYaaakiabg2da9iabdQeakjabdUealnaaqafabaGaeiikaGIaemywaK1aaSbaaSqaaiabdMgaPjabgkci3kabgkci3cqabaGccqGHsislaSqaaiabdMgaPbqab0GaeyyeIuoakiabdMfaznaaBaaaleaacqGHIaYTcqGHIaYTcqGHIaYTaeqaaOGaeiykaKYaaWbaaSqabeaacqaIYaGmaaaaaa@5D32@	JK∑αi2I−1+(KσP¯2+σE2) MathType@MTEF@5@5@+=feaafiart1ev1aaatCvAUfKttLearuWrP9MDH5MBPbIqV92AaeXatLxBI9gBaebbnrfifHhDYfgasaacH8akY=wiFfYdH8Gipec8Eeeu0xXdbba9frFj0=OqFfea0dXdd9vqai=hGuQ8kuc9pgc9s8qqaq=dirpe0xb9q8qiLsFr0=vr0=vr0dc8meaabaqaciaacaGaaeqabaqabeGadaaakeaadaWcaaqaaiabdQeakjabdUealnaaqaeabaacciGae8xSde2aa0baaSqaaiabdMgaPbqaaiabikdaYaaaaeqabeqdcqGHris5aaGcbaGaemysaKKaeyOeI0IaeGymaedaaiabgUcaRiabcIcaOiabdUealjab=n8aZnaaDaaaleaacuWGqbaugaqeaaqaaiabikdaYaaakiabgUcaRiab=n8aZnaaDaaaleaacqWGfbqraeaacqaIYaGmaaGccqGGPaqkaaa@44DA@
Pools nested in case or control (*P*)	2(*J *- 1)	∑i∑j∑k(Yij•−Yi••)2=K∑i∑j(Yij•−Yi••)2 MathType@MTEF@5@5@+=feaafiart1ev1aaatCvAUfKttLearuWrP9MDH5MBPbIqV92AaeXatLxBI9gBaebbnrfifHhDYfgasaacH8akY=wiFfYdH8Gipec8Eeeu0xXdbba9frFj0=OqFfea0dXdd9vqai=hGuQ8kuc9pgc9s8qqaq=dirpe0xb9q8qiLsFr0=vr0=vr0dc8meaabaqaciaacaGaaeqabaqabeGadaaakeaadaaeqbqaamaaqafabaWaaabuaeaacqGGOaakcqWGzbqwdaWgaaWcbaGaemyAaKMaemOAaOMaeyOiGClabeaakiabgkHiTaWcbaGaem4AaSgabeqdcqGHris5aaWcbaGaemOAaOgabeqdcqGHris5aaWcbaGaemyAaKgabeqdcqGHris5aOGaemywaK1aaSbaaSqaaiabdMgaPjabgkci3kabgkci3cqabaGccqGGPaqkdaahaaWcbeqaaiabikdaYaaakiabg2da9iabdUealnaaqafabaWaaabuaeaacqGGOaakcqWGzbqwdaWgaaWcbaGaemyAaKMaemOAaOMaeyOiGClabeaakiabgkHiTaWcbaGaemOAaOgabeqdcqGHris5aOGaemywaK1aaSbaaSqaaiabdMgaPjabgkci3kabgkci3cqabaGccqGGPaqkdaahaaWcbeqaaiabikdaYaaaaeaacqWGPbqAaeqaniabggHiLdaaaa@5EE6@	(KσP¯2+σE2) MathType@MTEF@5@5@+=feaafiart1ev1aaatCvAUfKttLearuWrP9MDH5MBPbIqV92AaeXatLxBI9gBaebbnrfifHhDYfgasaacH8akY=wiFfYdH8Gipec8Eeeu0xXdbba9frFj0=OqFfea0dXdd9vqai=hGuQ8kuc9pgc9s8qqaq=dirpe0xb9q8qiLsFr0=vr0=vr0dc8meaabaqaciaacaGaaeqabaqabeGadaaakeaacqGGOaakcqWGlbWsiiGacqWFdpWCdaqhaaWcbaGafmiuaaLbaebaaeaacqaIYaGmaaGccqGHRaWkcqWFdpWCdaqhaaWcbaGaemyraueabaGaeGOmaidaaOGaeiykaKcaaa@388D@
Replicates (*E*)	*IJ*(*K *- 1)	∑i∑j∑k(Yijk−Yij•)2 MathType@MTEF@5@5@+=feaafiart1ev1aaatCvAUfKttLearuWrP9MDH5MBPbIqV92AaeXatLxBI9gBaebbnrfifHhDYfgasaacH8akY=wiFfYdH8Gipec8Eeeu0xXdbba9frFj0=OqFfea0dXdd9vqai=hGuQ8kuc9pgc9s8qqaq=dirpe0xb9q8qiLsFr0=vr0=vr0dc8meaabaqaciaacaGaaeqabaqabeGadaaakeaadaaeqbqaamaaqafabaWaaabuaeaacqGGOaakcqWGzbqwdaWgaaWcbaGaemyAaKMaemOAaOMaem4AaSgabeaakiabgkHiTaWcbaGaem4AaSgabeqdcqGHris5aOGaemywaK1aaSbaaSqaaiabdMgaPjabdQgaQjabgkci3cqabaGccqGGPaqkdaahaaWcbeqaaiabikdaYaaaaeaacqWGQbGAaeqaniabggHiLdaaleaacqWGPbqAaeqaniabggHiLdaaaa@461F@	σE2 MathType@MTEF@5@5@+=feaafiart1ev1aaatCvAUfKttLearuWrP9MDH5MBPbIqV92AaeXatLxBI9gBaebbnrfifHhDYfgasaacH8akY=wiFfYdH8Gipec8Eeeu0xXdbba9frFj0=OqFfea0dXdd9vqai=hGuQ8kuc9pgc9s8qqaq=dirpe0xb9q8qiLsFr0=vr0=vr0dc8meaabaqaciaacaGaaeqabaqabeGadaaakeaaiiGacqWFdpWCdaqhaaWcbaGaemyraueabaGaeGOmaidaaaaa@30A8@

Abbreviations for column headings are as noted below.

DF: the degrees of freedom for the respective source row;

SS: the sum of squares for the respective source row;

E(MS): expectation of the mean square for the respective source row.

The sums of squares are based on the following terms:

Yij•=∑k=1KYijkK,Yi••=∑j=1J∑k=1KYijkJK, and Y•••=∑i=01∑j=1J∑k=1KYijk2JK.
 MathType@MTEF@5@5@+=feaafiart1ev1aaatCvAUfKttLearuWrP9MDH5MBPbIqV92AaeXatLxBI9gBaebbnrfifHhDYfgasaacH8akY=wiFfYdH8Gipec8Eeeu0xXdbba9frFj0=OqFfea0dXdd9vqai=hGuQ8kuc9pgc9s8qqaq=dirpe0xb9q8qiLsFr0=vr0=vr0dc8meaabaqaciaacaGaaeqabaqabeGadaaakeaacqWGzbqwdaWgaaWcbaGaemyAaKMaemOAaOMaeyOiGClabeaakiabg2da9maalaaabaWaaabCaeaacqWGzbqwdaWgaaWcbaGaemyAaKMaemOAaOMaem4AaSgabeaaaeaacqWGRbWAcqGH9aqpcqaIXaqmaeaacqWGlbWsa0GaeyyeIuoaaOqaaiabdUealbaacqGGSaalcqWGzbqwdaWgaaWcbaGaemyAaKMaeyOiGCRaeyOiGClabeaakiabg2da9maalaaabaWaaabCaeaadaaeWbqaaiabdMfaznaaBaaaleaacqWGPbqAcqWGQbGAcqWGRbWAaeqaaaqaaiabdUgaRjabg2da9iabigdaXaqaaiabdUealbqdcqGHris5aaWcbaGaemOAaOMaeyypa0JaeGymaedabaGaemOsaOeaniabggHiLdaakeaacqWGkbGscqWGlbWsaaGaeiilaWIaeeiiaaIaeeyyaeMaeeOBa4MaeeizaqMaeeiiaaIaemywaK1aaSbaaSqaaiabgkci3kabgkci3kabgkci3cqabaGccqGH9aqpdaWcaaqaamaaqahabaWaaabCaeaadaaeWbqaaiabdMfaznaaBaaaleaacqWGPbqAcqWGQbGAcqWGRbWAaeqaaaqaaiabdUgaRjabg2da9iabigdaXaqaaiabdUealbqdcqGHris5aaWcbaGaemOAaOMaeyypa0JaeGymaedabaGaemOsaOeaniabggHiLdaaleaacqWGPbqAcqGH9aqpcqaIWaamaeaacqaIXaqma0GaeyyeIuoaaOqaaiabikdaYiabdQeakjabdUealbaacqGGUaGlaaa@88A6@

The model we consider for individual pooled allele frequency estimates is

Yijk=μ+αi+Pj(i)+σEEijk=AijkAijk+Bijk,
 MathType@MTEF@5@5@+=feaafiart1ev1aaatCvAUfKttLearuWrP9MDH5MBPbIqV92AaeXatLxBI9gBaebbnrfifHhDYfgasaacH8akY=wiFfYdH8Gipec8Eeeu0xXdbba9frFj0=OqFfea0dXdd9vqai=hGuQ8kuc9pgc9s8qqaq=dirpe0xb9q8qiLsFr0=vr0=vr0dc8meaabaqaciaacaGaaeqabaqabeGadaaakeaacqWGzbqwdaWgaaWcbaGaemyAaKMaemOAaOMaem4AaSgabeaakiabg2da9GGaciab=X7aTjabgUcaRiab=f7aHnaaBaaaleaacqWGPbqAaeqaaOGaey4kaSIaemiuaa1aaSbaaSqaaiabdQgaQjabcIcaOiabdMgaPjabcMcaPaqabaGccqGHRaWkcqWFdpWCdaWgaaWcbaGaemyraueabeaakiabdweafnaaBaaaleaacqWGPbqAcqWGQbGAcqWGRbWAaeqaaOGaeyypa0ZaaSaaaeaacqWGbbqqdaWgaaWcbaGaemyAaKMaemOAaOMaem4AaSgabeaaaOqaaiabdgeabnaaBaaaleaacqWGPbqAcqWGQbGAcqWGRbWAaeqaaOGaey4kaSIaemOqai0aaSbaaSqaaiabdMgaPjabdQgaQjabdUgaRbqabaaaaOGaeiilaWcaaa@5BDA@

where the "group" effect associated with cases or controls is αi=E(Πi)−E(Π0+Π12)
 MathType@MTEF@5@5@+=feaafiart1ev1aaatCvAUfKttLearuWrP9MDH5MBPbIqV92AaeXatLxBI9gBaebbnrfifHhDYfgasaacH8akY=wiFfYdH8Gipec8Eeeu0xXdbba9frFj0=OqFfea0dXdd9vqai=hGuQ8kuc9pgc9s8qqaq=dirpe0xb9q8qiLsFr0=vr0=vr0dc8meaabaqaciaacaGaaeqabaqabeGadaaakeaaiiGacqWFXoqydaWgaaWcbaGaemyAaKgabeaakiabg2da9iabdweafjabcIcaOiabfc6aqnaaBaaaleaacqWGPbqAaeqaaOGaeiykaKIaeyOeI0IaemyrauKaeiikaGYaaSaaaeaacqqHGoaudaWgaaWcbaGaeGimaadabeaakiabgUcaRiabfc6aqnaaBaaaleaacqaIXaqmaeqaaaGcbaGaeGOmaidaaiabcMcaPaaa@4199@, *i *= 0,1 subject to the constraint ∑*α*_*i *_= 0. The random effect associated with the *j*th pool in either cases or controls is Pj(i)~N(0,σP,i2)
 MathType@MTEF@5@5@+=feaafiart1ev1aaatCvAUfKttLearuWrP9MDH5MBPbIqV92AaeXatLxBI9gBaebbnrfifHhDYfgasaacH8akY=wiFfYdH8Gipec8Eeeu0xXdbba9frFj0=OqFfea0dXdd9vqai=hGuQ8kuc9pgc9s8qqaq=dirpe0xb9q8qiLsFr0=vr0=vr0dc8meaabaqaciaacaGaaeqabaqabeGadaaakeaacqWGqbaudaWgaaWcbaGaemOAaOMaeiikaGIaemyAaKMaeiykaKcabeaakiabc6ha+jabd6eaojabcIcaOiabicdaWiabcYcaSGGaciab=n8aZnaaDaaaleaacqWGqbaucqGGSaalcqWGPbqAaeaacqaIYaGmaaGccqGGPaqkaaa@3EF5@, with σP,i2=Jτi2N
 MathType@MTEF@5@5@+=feaafiart1ev1aaatCvAUfKttLearuWrP9MDH5MBPbIqV92AaeXatLxBI9gBaebbnrfifHhDYfgasaacH8akY=wiFfYdH8Gipec8Eeeu0xXdbba9frFj0=OqFfea0dXdd9vqai=hGuQ8kuc9pgc9s8qqaq=dirpe0xb9q8qiLsFr0=vr0=vr0dc8meaabaqaciaacaGaaeqabaqabeGadaaakeaaiiGacqWFdpWCdaqhaaWcbaGaemiuaaLaeiilaWIaemyAaKgabaGaeGOmaidaaOGaeyypa0ZaaSaaaeaacqWGkbGscqWFepaDdaqhaaWcbaGaemyAaKgabaGaeGOmaidaaaGcbaGaemOta4eaaaaa@3A9F@. Finally, {*E*_*ijk*_} are independent *N*(0, 1) random variables and τi2
 MathType@MTEF@5@5@+=feaafiart1ev1aaatCvAUfKttLearuWrP9MDH5MBPbIqV92AaeXatLxBI9gBaebbnrfifHhDYfgasaacH8akY=wiFfYdH8Gipec8Eeeu0xXdbba9frFj0=OqFfea0dXdd9vqai=hGuQ8kuc9pgc9s8qqaq=dirpe0xb9q8qiLsFr0=vr0=vr0dc8meaabaqaciaacaGaaeqabaqabeGadaaakeaaiiGacqWFepaDdaqhaaWcbaGaemyAaKgabaGaeGOmaidaaaaa@30F2@ is the variance of the allele frequency in the *i*^th ^group.

The power calculation of the *F*-test, the standard statistical procedure used when testing allele frequency differences for DNA pooling, requires the non-centrality parameter (NCP) of the test. Its approximate value is given in equation 1 of the Methods and Technical Issues section below. The NCP is a function of the difference between the case and control allele 2 frequencies, the quality of the pooling estimate of these probabilities, the number of cases and controls, the number of replications of DNA measurements of each pool, and the size of each pool.

When the number of replicates *K *is fixed, the approximate NCP is constant with respect to the number of pools (*J*). When the number of pools *J *is larger, the denominator degrees of freedom (df) are larger, so that the power of the *F*-test is greater. That is, smaller pool sizes *T *= *N/J *for larger *J*, have greater power. The protocol of genotyping each subject has *T *= 1, which is the most powerful allele frequency testing protocol. That is, if genotype cost is not an issue, it is always most powerful to individually genotype all subjects.

**Figure 1:**
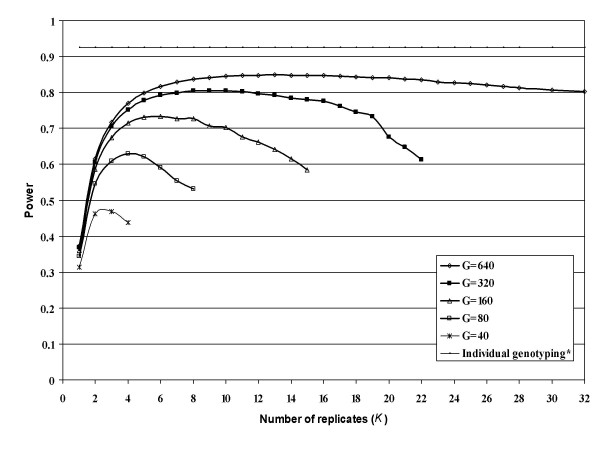
**Power as a function of number of replicates (*K*) for fixed number of genotypings (*G *= *J *× *K*) with recessive mode of inheritance**. Power values presented here are for studies with *N *= 10000, prevalence *φ *= 0.05, disease allele frequency *p*_*d *_= 0.15, relative risk of homozygous for disease allele *R*_2 _= 3, minor SNP marker allele frequency *q*_1 _= 0.35, machine replicability variance factor *m *= 2.25, linkage disequilibrium pr=0.9Rmax⁡2
 MathType@MTEF@5@5@+=feaafiart1ev1aaatCvAUfKttLearuWrP9MDH5MBPbIqV92AaeXatLxBI9gBaebbnrfifHhDYfgasaacH8akY=wiFfYdH8Gipec8Eeeu0xXdbba9frFj0=OqFfea0dXdd9vqai=hGuQ8kuc9pgc9s8qqaq=dirpe0xb9q8qiLsFr0=vr0=vr0dc8meaabaqaciaacaGaaeqabaqabeGadaaakeaacqWGWbaCdaWgaaWcbaGaemOCaihabeaakiabg2da9iabicdaWiabc6caUiabiMda5iabdkfasnaaDaaaleaacyGGTbqBcqGGHbqycqGG4baEaeaacqaIYaGmaaaaaa@3A02@ and significance level alpha = 0.0001. *The horizontal line represents the power for specified parameters with individual genotyping using the 2 × 2 test of independence. Power with individual genotyping was computed using the method implemented in the Power for Association With Error (PAWE) website [27].

When the total number of genotypings (*G *= *J *× *K*) is fixed, as is the situation for a fixed budget, the optimal choice of *J *and *K *is more complex. When one knows the genetic model parameters, one can examine the power using a range of values of *J *and *K *(and hence *T*) to find settings with high power. We seek to find *K*_*o*_(*G*), the number of replicates that has greatest power when there are *G *genotypings. For example, Figure 1 is based on a recessive mode of inheritance (MOI) with *N *= 10,000, prevalence *φ *= 0.05, disease allele frequency *p*_*d *_= 0.15, relative risk of homozygous for disease allele (*R*_2_) is 3, linkage disequilibrium pr=0.9Rmax⁡2
 MathType@MTEF@5@5@+=feaafiart1ev1aaatCvAUfKttLearuWrP9MDH5MBPbIqV92AaeXatLxBI9gBaebbnrfifHhDYfgasaacH8akY=wiFfYdH8Gipec8Eeeu0xXdbba9frFj0=OqFfea0dXdd9vqai=hGuQ8kuc9pgc9s8qqaq=dirpe0xb9q8qiLsFr0=vr0=vr0dc8meaabaqaciaacaGaaeqabaqabeGadaaakeaacqWGWbaCdaWgaaWcbaGaemOCaihabeaakiabg2da9iabicdaWiabc6caUiabiMda5iabdkfasnaaDaaaleaacyGGTbqBcqGGHbqycqGG4baEaeaacqaIYaGmaaaaaa@3A02@, (where Rmax⁡2
 MathType@MTEF@5@5@+=feaafiart1ev1aaatCvAUfKttLearuWrP9MDH5MBPbIqV92AaeXatLxBI9gBaebbnrfifHhDYfgasaacH8akY=wiFfYdH8Gipec8Eeeu0xXdbba9frFj0=OqFfea0dXdd9vqai=hGuQ8kuc9pgc9s8qqaq=dirpe0xb9q8qiLsFr0=vr0=vr0dc8meaabaqaciaacaGaaeqabaqabeGadaaakeaacqWGsbGudaqhaaWcbaGagiyBa0MaeiyyaeMaeiiEaGhabaGaeGOmaidaaaaa@331E@ is the maximum disequilibrium value between the disease allele and the coupling SNP allele; also see PAWE-3D website Helpfile [19], minor SNP allele frequency *q*_1 _= 0.35, and machine replicability variance factor *m *= 2.25. We set *G *= *J *× *K *to 80, 160, 320, and 640 with significance level 0.0001. The power increases substantially as *G *increases. For example, the maximum power is 0.73 with 80 genotypings when *K*_*o*_(80) = 4; that is, 4 replicates of each of 20 pools. It increases to 0.91 with 640 genotypings when *K*_*o*_(640) = 13; that is 13 replicates each of 49 pools. The power of the chi-squared 2 × 2 test of independence when each subject is individually genotyped is 0.97. With 640 genotypings, the power with *K *= 4 is 0.85. The increase of power from 0.73 to 0.91 is obtained through additional genotyping effort rather than increased sampling of subjects. Also note that the power when *K *= 1 is always substantially less than the power using the optimal choice of *K*; that is, replication of pool measurement is always advantageous.

**Figure 2:**
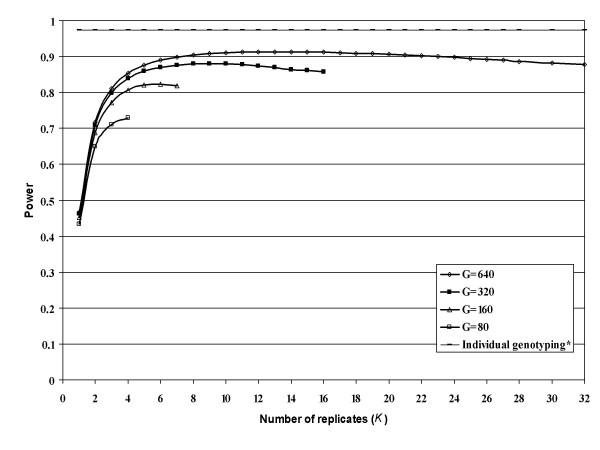
**Power as a function of number of replicates (*K*) for fixed number of genotypings (*G *= *J *× *K*) with dominant mode of inheritance**. Power values presented here are for studies with *N *= 5000, prevalence *φ *= 0.05, disease allele frequency *p*_*d *_= 0.15, relative risk of a genotype with at least one copy of the disease allele = 1.5, minor SNP marker allele frequency *q*_1 _= 0.35, machine replicability variance factor *m *= 2.25, linkage disequilibrium pr=0.9Rmax⁡2
 MathType@MTEF@5@5@+=feaafiart1ev1aaatCvAUfKttLearuWrP9MDH5MBPbIqV92AaeXatLxBI9gBaebbnrfifHhDYfgasaacH8akY=wiFfYdH8Gipec8Eeeu0xXdbba9frFj0=OqFfea0dXdd9vqai=hGuQ8kuc9pgc9s8qqaq=dirpe0xb9q8qiLsFr0=vr0=vr0dc8meaabaqaciaacaGaaeqabaqabeGadaaakeaacqWGWbaCdaWgaaWcbaGaemOCaihabeaakiabg2da9iabicdaWiabc6caUiabiMda5iabdkfasnaaDaaaleaacyGGTbqBcqGGHbqycqGG4baEaeaacqaIYaGmaaaaaa@3A02@ and significance level alpha = 0.0001. *The horizontal line represents the power for specified parameters with individual genotyping using the 2 × 2 test of independence. Power with individual genotyping was computed using the method implemented in the Power for Association With Error (PAWE) website [27].

Figure 2 is based on a dominant MOI with *N *= 5,000, prevalence *φ *= 0.05, disease allele frequency *p*_*d *_= 0.15, relative risk of a genotype with at least one copy of the disease allele is 1.5, linkage disequilibrium pr=0.9Rmax⁡2
 MathType@MTEF@5@5@+=feaafiart1ev1aaatCvAUfKttLearuWrP9MDH5MBPbIqV92AaeXatLxBI9gBaebbnrfifHhDYfgasaacH8akY=wiFfYdH8Gipec8Eeeu0xXdbba9frFj0=OqFfea0dXdd9vqai=hGuQ8kuc9pgc9s8qqaq=dirpe0xb9q8qiLsFr0=vr0=vr0dc8meaabaqaciaacaGaaeqabaqabeGadaaakeaacqWGWbaCdaWgaaWcbaGaemOCaihabeaakiabg2da9iabicdaWiabc6caUiabiMda5iabdkfasnaaDaaaleaacyGGTbqBcqGGHbqycqGG4baEaeaacqaIYaGmaaaaaa@3A02@, minor SNP allele frequency *q*_1 _= 0.35, and machine replicability variance factor *m *= 2.25. We set the number of genotypings *J *× *K *to 40, 80, 160, 320, and 640. The pattern is similar to that of Figure 1. A program is available from the corresponding author to produce these numbers for user specified settings.

We note that, although results are not presented, we performed analyses similar to those presented in Figures 1 and 2 for a multiplicative MOI. The conclusions were the same, with results being very similar to the dominant MOI results (Figure 2). We omit these results in the interest of brevity.

The program mentioned above was  used to create Table 2, which considers the robustness of design choices when studying a disease with prevalence equal to 0.05. We consider both dominant and recessive MOI with genetic relative risk (GRR) values ranging from 1.5 to 2.2 for specified levels of significance, linkage disequilibrium, sample size, minor SNP marker allele frequency and quality of pooling measurement *m*. We examine the range of numbers of genotypings *J *× *K *between 40 and 640. Table 2 gives the maximum power for each number of genotypings, *K*_*o*_(*G*), and the range of *K *settings that produce power within 95% of the maximal power. As in Figures 1 and 2, the most important result is that increasing *G *always substantially increases power. For example, in scenario 1 with 10,000 subjects in each group, recessive MOI, relative risk 2.2, and level of significance 0.0001, the maximal power is 38% with 40 genotypings compared to 77% with 640 genotypings. Similar patterns hold for the other situations considered. The value of *K*_*o*_(*G*) increases at a less than linear rate as *G *increases. Typically, the decrease in power associated with using a value of *K *slightly different from *K*_*o*_(*G*) is relatively small; that is, the power of the procedure is relatively insensitive to choice of *K*. While *K *= 4 is optimal or close to optimal when the number of genotypings is small (i.e. *G *= 40 or 80), *K*_*o*_(*G*) increases with *G *and can have appreciable greater power than with 4 replicates. The value of *K*_*o*_(*G*) is not substantially affected by whether the MOI is dominant (see scenarios 8–10) or recessive (see scenarios 1–7).

**Table 2:** Maximum power as a function of the number of genotyping(*G *= *J *× *K*), number of replicates giving maximum power (*K*_*o*_(*G*)), number of replicates (*K*) at 95% of the maximum power at specific experimental and genetic parameters and the power at *K *= 1 when assuming no machine replicability variability (*m *= 1)

Situation	*N*	MOI	*R*_2_	*α*	*m*	*MAF*	*p*_*r*_	G = 40	G = 80	G = 160	G = 320	G = 640
1	10000	R	2.2	0.0001	2.25	0.20	0.9	38%, 2, (2), 82%	54%, 4, (3–4), 85%	64%,6, (4–7), 87%	72%, 10, (5–16), 87%	77%, 13, (6–27), 88%
2	10000	R	2.0	0.001	2.25	0.20	0.9	43%, 2, (2), 79%	56%, 4, (3–4), 81%	65%, 7, (4–7), 82%	71%, 11, (6–16), 83%	75%, 16, (7–32), 83%
3	10000	R	1.8	0.01	2.25	0.20	0.9	50%, 2, (2), 79%	62%, 4, (3–4), 80%	68%, 7, (5–7), 80%	72%, 13, (6–16), 80%	75%, 20, (7–32), 81%
4	10000	R	2.2	0.0001	2.25	0.15	1	69%, 2, (2), 98%	84%, 4, (3–4), 99%	90%, 6, (3–7), 99%	95%, 10, (4–16), 99%	96%, 13, (4–32), 99%
5	10000	R	2.2	0.0001	2.0	0.15	1	75%, 2, (2), 98%	87%, 4, (2–4), 99%	92%, 5, (3–7), 99%	95%, 8, (3–16), 99%	97%, 12 (3–32), 99%
6	10000	R	2.0	0.0001	2.25	0.15	1	43%,2, (2), 86%	59%, 4, (3–4), 89%	70%, 6, (4–7), 90%	77%, 10, (5–16), 91%	82%, 13, (6–28), 91%
7	10000	R	2.0	0.0001	2.0	0.15	1	49%, 2 (2), 86%	63%, 4, (3–4), 89%	73%, 5, (4–7), 90%	79%, 8, (4–16), 91%	83%, 12, (5–27), 91%
8	2000	D	1.5	0.0001	2.25	0.15	0.9	57%, 3, (2–3), 94%	73%, 4, (3–6), 96%	82%, 6, (4–10), 96%	88%, 10, (4–18), --	91%, 13, (5–32), --
9	2000	D	1.5	0.0001	2.25	0.15	1	65%, 3, (2–3), 97%	80%, 4, (3–6),98%	88%, 6, (3–9), 98	92%, 10, (4–21), --	95%, 13, (4–40), --
10	2000	D	1.5	0.0001	2.0	0.15	1	70%, 2, (2–3), 97%	83%, 4, (3–6), 98%	90%, 5. (3–10), 98%	94%, 8, (3–20), --	96%, 12, (4–37), --

We only consider designs in which the pool size (*T*) is between 10 and 500. The prevalence *φ *is 0.05 and disease allele frequency *p*_*d *_is 0.15.

--: the size of pool is out of our consideration, no power is provided;

*N*: sample size in cases or controls;

MOI: mode of inheritance (R = recessive MOI and D= dominant MOI);

*R*_2_: relative risk for subjects homozygous for disease allele;

*α*: significance level;

*m*: machine replicability variance factor;

*MAF*: minor SNP marker allele frequency;

*p*_*r*_: measure of linkage disequilibrium.

### Regression modeling results

We use ordinary least squares (OLS) regression analysis with power at the 0.0001 significance level as the dependent variable for each of the 4^4 ^× 2^3 ^× 3 × 5 (30720) model specifications. We consider the 9 factors listed in Table 3 and all possible two-way combinations in our regression model to assess the relative importance of the factors in determination of power to detect association. We also use the square of the number of replicates to model the optimal number of replicates. The analysis finds a significant fit (*F*_55,30664 _= 1348.07, p-value < 0.0001) with *R*^2 ^equal to 0.71. Genotype relative risk (*R*_2_) has the largest *F*-statistic (34333.5 with 1 df), with increasing *R*_2 _associated with greater power. Sample size has the second largest *F*-statistic (15002.4 with 1 df). The MOI also has a highly significant *F*-statistic (5869.7 with 2 df). For a fixed genotype relative risk *R*_2, _the median power is greatest for dominant MOI, followed by multiplicative and then recessive MOIs. The prevalence of the disease (*φ*), the minor marker allele frequency, and the measurement quality of the pooling are the factors that have the smallest *F*-statistic values. Measurement error explains less of the variance than genetic parameters. In general, increased measurement error reduces the power of the procedure. Further, with genetic parameters fixed, the decrease in power from increased measurement error can be offset either by an increase in *K *or decrease of the number of individuals in each pool.

**Table 3:** List of parameters considered in the multiple regression analysis

Parameter	Description	Value
*N*	Number of case (control) subjects	1000, 2000, 5000, 10000
*φ*	Prevalence of the disease	0.01, 0.1
*T*	Size of the pool	25, 50, 100, 250, 500
*K*	Number of replicates of each pool	1, 2, 4, 8
*p*_*d*_	Disease allele frequency	0.1, 0.25
MOI	Modes of inheritance	dominant, recessive, multiplicative
*R*_2_	Genotype relative risk of homozygote of disease allele	*1.2, 1.5, 2.25, 4
*q*_1_	Minor SNP marker allele frequency	0.1, 0.35
*M*	Machine replicability variance factor	2.05, 2.1, 2.25, 3

*R_1 _is obtained according to the relationship between *R*_1 _and *R*_2_; that is for multiplicative MOI, *R*_2 _= R12
 MathType@MTEF@5@5@+=feaafiart1ev1aaatCvAUfKttLearuWrP9MDH5MBPbIqV92AaeXatLxBI9gBaebbnrfifHhDYfgasaacH8akY=wiFfYdH8Gipec8Eeeu0xXdbba9frFj0=OqFfea0dXdd9vqai=hGuQ8kuc9pgc9s8qqaq=dirpe0xb9q8qiLsFr0=vr0=vr0dc8meaabaqaciaacaGaaeqabaqabeGadaaakeaacqWGsbGudaqhaaWcbaGaeGymaedabaGaeGOmaidaaaaa@2FE8@; dominant MOI, *R*_1 _= *R*_2_; recessive MOI, *R*_1 _= 1. We considered all 30720 (4^4 ^× 2^3 ^× 3 × 5) situations generated from the parameters listed above.

Among the interaction terms not involving *K*, *p*_*d *_× *q*_1_, *p*_*d *_× *MOI*, *R*_2 _× *T*, *p*_*d *_× *R*_2_, and *N *× *T *are highly significant (sorted in increasing *P*-values). The most significant interaction term is *p*_*d *_× *q*_1_. This finding is not surprising as there has been extensive documentation in the statistical genetics literature that power for genetic association is maximized when the difference between the disease allele frequency and the SNP marker allele frequency in coupling with the disease allele is 0, with decreasing power occurring as the difference increases [20-23]. The finding of a significant interaction *p*_*d *_× *MOI *between disease allele frequency and disease MOI has also been documented previously, most recently in the work by Skol et al. [4]. The finding underscores the fact that, when all other factors are fixed, the disease allele frequency that gives optimal power differs depending upon the disease MOI.

## Discussion

Our results have produced two types of conclusions. The first is that the genetic parameters of the disease being studied are the most important determinants of the power to detect association. This fact is consistent with the association of ApoE with late onset Alzheimer's Disease [24] and recent association results for age-related macular degeneration [1,3]. In each of these studies, estimated genotype relative risks are approximately 3 for the heterozygote and greater than 9 for the homozygote. In all studies, highly significant associations were observed with less than 500 total cases and controls. Furthermore, for age-related macular degeneration [24], associations were observed for SNP alleles in linkage disequilibrium (LD) with the functional variants. The results from the OLS regression analysis are consistent with this history. The genetic relative risk is the most significant parameter, followed by the sample size. For a fixed genotype relative risk *R*_2, _the median power is greatest for dominant MOI, followed by multiplicative and then recessive MOIs. The linear and quadratic terms in the number of replicates *K *and a number of interactions with *K *are significant. Since there is an optimal setting of *K*, this result is expected.

The second type of conclusion is guidance about the choice of the number of genotypings *G *= *J *× *K *and the simultaneous setting of the number of replicates *K *of the *J *pools. We have shown that the number of genotypings *G *= *J *× *K *should be as large as possible (holding all other factors constant) to have the greatest power. When *G *is fixed, we have shown that there is a setting *K*_*o*_(*G*) that maximizes the power when all genetic model parameters are specified. The optimal setting increases as *G *increases. These differences are practically important and suggest that those conducting pooled studies use the program available from the corresponding author to determine optimal settings. In all situations studied, for fixed value of *G*, power is relatively insensitive to choice of *K *near *K*_*o*_(*G*). Further, when the machine replicability variance factor *m *is larger than 1, the setting *K *= 1 has power much less than replicated designs. This suggests that such extensions of these designs as staggered nested designs [18] may have little value in genetic pooling studies.

Our work provides the basis for extending recommendations such as those of Sham et al. [10] to include genetic model parameters. For the very large studies possible with pooling, there is strong evidence that increasing the number of genotypings and increasing the number of replicate measurements of each pool can increase power noticeably. This approach is dependent on the assumption that *E*(Π_*i*_) = *E*(*Y*_*ijk*_), where Π_*i *_is the fraction of the major allele 2 in a randomly selected subject from the *i*^th ^group; that is, the pooled estimate of the intensity of an allele is in fact an unbiased estimate of the allele 2 frequency. Further work will incorporate designs that formally include validation of this assumption.

## Conclusion

Our work extends that of previous researchers who have considered power and sample size calculations for genetic association studies with pooled DNA samples (e.g., [16]). Our extension involves inclusion of genetic model parameters such as disease MOI, disease allele frequency, disease prevalence, marker allele frequency, and genotype relative risks. It is clear from the results of our regression analysis that incorporation of such parameters is important in the design of more powerful genetic association tests. We recommend that researchers incorporate information into their power and sample size calculations for genetic association with pooled DNA, such as choice of numbers of genotypings and the number of replicates that can increase power from such relatively low levels as 40% to 50% to 75% to 80% using the same cases and controls.

## Methods

### Definitions

*N*: number of case (control) subjects; we assume equal numbers of cases and controls (balanced design).

*J*: number of pools; *J *≥ 2.

*T = N/J*: number of individuals in each pool; we assume that case subjects are assigned randomly to case pools and control subjects are assigned randomly to control pools.

*K*: number of replicates of each pool; we assume that there is no reassignment of subjects in the replications.

*G *= *J *× *K*: number of case (control) genotypings.

### Genetic model parameters

We consider a disease associated with a di-allelic gene with allele *d *associated with increased risk of disease and allele + associated with no increased risk.

*p*_*d*_: allele frequency of disease locus *d *allele.

*p*_+ _= 1 - *p*_*d*_: allele frequency of disease locus wild-type (+) allele.

*φ*: prevalence of the disease.

*f*_2_: probability of having disease with 2 disease alleles in the genotype = penetrance of *dd*.

*f*_1_: probability of having disease with 1 disease allele in the genotype = penetrance of *d*+.

*f*_0_: probability of having disease with 0 disease alleles in the genotype = penetrance of ++.

### Genotype relative risks (GRR)

R2=f2f0.
 MathType@MTEF@5@5@+=feaafiart1ev1aaatCvAUfKttLearuWrP9MDH5MBPbIqV92AaeXatLxBI9gBaebbnrfifHhDYfgasaacH8akY=wiFfYdH8Gipec8Eeeu0xXdbba9frFj0=OqFfea0dXdd9vqai=hGuQ8kuc9pgc9s8qqaq=dirpe0xb9q8qiLsFr0=vr0=vr0dc8meaabaqaciaacaGaaeqabaqabeGadaaakeaacqWGsbGudaWgaaWcbaGaeGOmaidabeaakiabg2da9maalaaabaGaemOzay2aaSbaaSqaaiabikdaYaqabaaakeaacqWGMbGzdaWgaaWcbaGaeGimaadabeaaaaGccqGGUaGlaaa@35F1@

R1=f1f0.
 MathType@MTEF@5@5@+=feaafiart1ev1aaatCvAUfKttLearuWrP9MDH5MBPbIqV92AaeXatLxBI9gBaebbnrfifHhDYfgasaacH8akY=wiFfYdH8Gipec8Eeeu0xXdbba9frFj0=OqFfea0dXdd9vqai=hGuQ8kuc9pgc9s8qqaq=dirpe0xb9q8qiLsFr0=vr0=vr0dc8meaabaqaciaacaGaaeqabaqabeGadaaakeaacqWGsbGudaWgaaWcbaGaeGymaedabeaakiabg2da9maalaaabaGaemOzay2aaSbaaSqaaiabigdaXaqabaaakeaacqWGMbGzdaWgaaWcbaGaeGimaadabeaaaaGccqGGUaGlaaa@35ED@

### Modes of Inheritance (MOI)

The three MOIs are characterized by the parameter *R*.

Multiplicative MOI: The penetrances satisfy the equation R=f1f0=f2f1
 MathType@MTEF@5@5@+=feaafiart1ev1aaatCvAUfKttLearuWrP9MDH5MBPbIqV92AaeXatLxBI9gBaebbnrfifHhDYfgasaacH8akY=wiFfYdH8Gipec8Eeeu0xXdbba9frFj0=OqFfea0dXdd9vqai=hGuQ8kuc9pgc9s8qqaq=dirpe0xb9q8qiLsFr0=vr0=vr0dc8meaabaqaciaacaGaaeqabaqabeGadaaakeaacqWGsbGucqGH9aqpdaWcaaqaaiabdAgaMnaaBaaaleaacqaIXaqmaeqaaaGcbaGaemOzay2aaSbaaSqaaiabicdaWaqabaaaaOGaeyypa0ZaaSaaaeaacqWGMbGzdaWgaaWcbaGaeGOmaidabeaaaOqaaiabdAgaMnaaBaaaleaacqaIXaqmaeqaaaaaaaa@39E7@; that is, R2=R12
 MathType@MTEF@5@5@+=feaafiart1ev1aaatCvAUfKttLearuWrP9MDH5MBPbIqV92AaeXatLxBI9gBaebbnrfifHhDYfgasaacH8akY=wiFfYdH8Gipec8Eeeu0xXdbba9frFj0=OqFfea0dXdd9vqai=hGuQ8kuc9pgc9s8qqaq=dirpe0xb9q8qiLsFr0=vr0=vr0dc8meaabaqaciaacaGaaeqabaqabeGadaaakeaacqWGsbGudaWgaaWcbaGaeGOmaidabeaakiabg2da9iabdkfasnaaDaaaleaacqaIXaqmaeaacqaIYaGmaaaaaa@3343@.

Dominant MOI: *R *= *R*_1 _= *R*_2_.

Recessive MOI: R=R2=R2R1
 MathType@MTEF@5@5@+=feaafiart1ev1aaatCvAUfKttLearuWrP9MDH5MBPbIqV92AaeXatLxBI9gBaebbnrfifHhDYfgasaacH8akY=wiFfYdH8Gipec8Eeeu0xXdbba9frFj0=OqFfea0dXdd9vqai=hGuQ8kuc9pgc9s8qqaq=dirpe0xb9q8qiLsFr0=vr0=vr0dc8meaabaqaciaacaGaaeqabaqabeGadaaakeaacqWGsbGucqGH9aqpcqWGsbGudaWgaaWcbaGaeGOmaidabeaakiabg2da9maalaaabaGaemOuai1aaSbaaSqaaiabikdaYaqabaaakeaacqWGsbGudaWgaaWcbaGaeGymaedabeaaaaaaaa@36E8@; that is, *R*_1 _= 1.

### SNP marker parameters

*q*_1_: allele frequency of minor SNP marker allele 1 (that is, 0 <*q*_1_≤ 0.5).

*q*_2_: the frequency of the major SNP marker allele 2.

### Disequilibrium parameters

*D*_max _= min(*p*_*d*_*q*_2_, *p*_+_*q*_1_).

Rmax⁡2=Dmax⁡2pdp+q1q2
 MathType@MTEF@5@5@+=feaafiart1ev1aaatCvAUfKttLearuWrP9MDH5MBPbIqV92AaeXatLxBI9gBaebbnrfifHhDYfgasaacH8akY=wiFfYdH8Gipec8Eeeu0xXdbba9frFj0=OqFfea0dXdd9vqai=hGuQ8kuc9pgc9s8qqaq=dirpe0xb9q8qiLsFr0=vr0=vr0dc8meaabaqaciaacaGaaeqabaqabeGadaaakeaacqWGsbGudaqhaaWcbaGagiyBa0MaeiyyaeMaeiiEaGhabaGaeGOmaidaaOGaeyypa0ZaaSaaaeaacqWGebardaqhaaWcbaGagiyBa0MaeiyyaeMaeiiEaGhabaGaeGOmaidaaaGcbaGaemiCaa3aaSbaaSqaaiabdsgaKbqabaGccqWGWbaCdaWgaaWcbaGaey4kaScabeaakiabdghaXnaaBaaaleaacqaIXaqmaeqaaOGaemyCae3aaSbaaSqaaiabikdaYaqabaaaaaaa@4529@ (see, e.g., [25]).

*p*_*r*_: measure of linkage disequilibrium between disease gene and SNP marker; here it is a fraction of Rmax⁡2
 MathType@MTEF@5@5@+=feaafiart1ev1aaatCvAUfKttLearuWrP9MDH5MBPbIqV92AaeXatLxBI9gBaebbnrfifHhDYfgasaacH8akY=wiFfYdH8Gipec8Eeeu0xXdbba9frFj0=OqFfea0dXdd9vqai=hGuQ8kuc9pgc9s8qqaq=dirpe0xb9q8qiLsFr0=vr0=vr0dc8meaabaqaciaacaGaaeqabaqabeGadaaakeaacqWGsbGudaqhaaWcbaGagiyBa0MaeiyyaeMaeiiEaGhabaGaeGOmaidaaaaa@331E@ (0 <*p*_*r *_≤ 1); the examples use *p*_*r *_= 0.9.

The detailed computation of case and control genotype probabilities which are functions of the disease allele frequency, minor SNP allele frequency, and linkage disequilibrium parameters are documented in the PAWE-3D Helpfile [19].

We use method [26] implemented in the PAWE software [27] to calculate the power of the 2 × 2 test of independence when each subject is individually genotyped and we report these value in Figures 1 and 2.

### Case-control frequency of allele 2

Π_*i*_: the fraction of the major allele 2 in a randomly selected subject from the *i*^th ^group, *i *= 0 for cases, *i *= 1 for controls. It follows that the expectation of Π_*i *_is given by:

E(Πi)=12Pi1+Pi2,
 MathType@MTEF@5@5@+=feaafiart1ev1aaatCvAUfKttLearuWrP9MDH5MBPbIqV92AaeXatLxBI9gBaebbnrfifHhDYfgasaacH8akY=wiFfYdH8Gipec8Eeeu0xXdbba9frFj0=OqFfea0dXdd9vqai=hGuQ8kuc9pgc9s8qqaq=dirpe0xb9q8qiLsFr0=vr0=vr0dc8meaabaqaciaacaGaaeqabaqabeGadaaakeaacqWGfbqrcqGGOaakcqqHGoaudaWgaaWcbaGaemyAaKgabeaakiabcMcaPiabg2da9maalaaabaGaeGymaedabaGaeGOmaidaaiabdcfaqnaaBaaaleaacqWGPbqAcqaIXaqmaeqaaOGaey4kaSIaemiuaa1aaSbaaSqaaiabdMgaPjabikdaYaqabaGccqGGSaalaaa@3E90@

where *P*_*i*1 _is the frequency of the heterozygous genotype with allele 2 in the *i*^th ^group and *P*_*i*2 _is the frequency of the homozygous genotype with allele 2 in the *i*^th ^group. In addition,

var⁡(Πi)=τi2=E(Πi2)−[E(Πi)]2=14Pi1(1−Pi1)+Pi2(1−Pi2)−Pi1Pi2.
 MathType@MTEF@5@5@+=feaafiart1ev1aaatCvAUfKttLearuWrP9MDH5MBPbIqV92AaeXatLxBI9gBaebbnrfifHhDYfgasaacH8akY=wiFfYdH8Gipec8Eeeu0xXdbba9frFj0=OqFfea0dXdd9vqai=hGuQ8kuc9pgc9s8qqaq=dirpe0xb9q8qiLsFr0=vr0=vr0dc8meaabaqaciaacaGaaeqabaqabeGadaaakeaacyGG2bGDcqGGHbqycqGGYbGCcqGGOaakcqqHGoaudaWgaaWcbaGaemyAaKgabeaakiabcMcaPiabg2da9GGaciab=r8a0naaDaaaleaacqWGPbqAaeaacqaIYaGmaaGccqGH9aqpcqWGfbqrcqGGOaakcqqHGoaudaqhaaWcbaGaemyAaKgabaGaeGOmaidaaOGaeiykaKIaeyOeI0Iaei4waSLaemyrauKaeiikaGIaeuiOda1aaSbaaSqaaiabdMgaPbqabaGccqGGPaqkcqGGDbqxdaahaaWcbeqaaiabikdaYaaakiabg2da9maalaaabaGaeGymaedabaGaeGinaqdaaiabdcfaqnaaBaaaleaacqWGPbqAcqaIXaqmaeqaaOGaeiikaGIaeGymaeJaeyOeI0Iaemiuaa1aaSbaaSqaaiabdMgaPjabigdaXaqabaGccqGGPaqkcqGHRaWkcqWGqbaudaWgaaWcbaGaemyAaKMaeGOmaidabeaakiabcIcaOiabigdaXiabgkHiTiabdcfaqnaaBaaaleaacqWGPbqAcqaIYaGmaeqaaOGaeiykaKIaeyOeI0Iaemiuaa1aaSbaaSqaaiabdMgaPjabigdaXaqabaGccqWGqbaudaWgaaWcbaGaemyAaKMaeGOmaidabeaakiabc6caUaaa@6FF5@

### Analysis of variance (ANOVA) table for two-stage nested design

#### Specification of ANOVA model

*A*_*ijk*_: intensity level of allele 2 in the *i*^th ^group (*i *= 0 for cases, 1 for controls), *j*^th ^pool (*j *= 1,...,*J*), *k*^th ^replicate (*k *= 1,...,*K*).

*B*_*ijk*_: intensity level of allele 1 in the *i*^th ^group (*i *= 0 for cases, 1 for controls), *j*^th ^pool (*j *= 1,..., *J*), *k*^th ^replicate (*k *= 1,..., *K*).

Yijk=AijkAijk+Bijk
 MathType@MTEF@5@5@+=feaafiart1ev1aaatCvAUfKttLearuWrP9MDH5MBPbIqV92AaeXatLxBI9gBaebbnrfifHhDYfgasaacH8akY=wiFfYdH8Gipec8Eeeu0xXdbba9frFj0=OqFfea0dXdd9vqai=hGuQ8kuc9pgc9s8qqaq=dirpe0xb9q8qiLsFr0=vr0=vr0dc8meaabaqaciaacaGaaeqabaqabeGadaaakeaacqWGzbqwdaWgaaWcbaGaemyAaKMaemOAaOMaem4AaSgabeaakiabg2da9maalaaabaGaemyqae0aaSbaaSqaaiabdMgaPjabdQgaQjabdUgaRbqabaaakeaacqWGbbqqdaWgaaWcbaGaemyAaKMaemOAaOMaem4AaSgabeaakiabgUcaRiabdkeacnaaBaaaleaacqWGPbqAcqWGQbGAcqWGRbWAaeqaaaaaaaa@442C@: fraction of SNP allele 2 estimated in the *i*^th ^group (i = 0 for cases, 1 for controls), *j*^th ^pool (*j *= 1,..., *J*), *k*^th ^replicate (*k *= 1,..., *K*).

Model:

*Y*_*ijk *_= *μ *+ *α*_*i *_+ *P*_*j*(*i*) _+ *σ*_*E*_*E*_*ijk*_,

where the case or control effect is αi=E(Πi)−E(Π0+Π12)
 MathType@MTEF@5@5@+=feaafiart1ev1aaatCvAUfKttLearuWrP9MDH5MBPbIqV92AaeXatLxBI9gBaebbnrfifHhDYfgasaacH8akY=wiFfYdH8Gipec8Eeeu0xXdbba9frFj0=OqFfea0dXdd9vqai=hGuQ8kuc9pgc9s8qqaq=dirpe0xb9q8qiLsFr0=vr0=vr0dc8meaabaqaciaacaGaaeqabaqabeGadaaakeaaiiGacqWFXoqydaWgaaWcbaGaemyAaKgabeaakiabg2da9iabdweafjabcIcaOiabfc6aqnaaBaaaleaacqWGPbqAaeqaaOGaeiykaKIaeyOeI0IaemyrauKaeiikaGYaaSaaaeaacqqHGoaudaWgaaWcbaGaeGimaadabeaakiabgUcaRiabfc6aqnaaBaaaleaacqaIXaqmaeqaaaGcbaGaeGOmaidaaiabcMcaPaaa@4199@, *i *= 0,1, subject to the constraint ∑*α*_*i *_= 0. The random sampling effect of the allele 2 frequency associated with the *j*^th ^pool in either cases or controls is Pj(i)~N(0,σP,i2)
 MathType@MTEF@5@5@+=feaafiart1ev1aaatCvAUfKttLearuWrP9MDH5MBPbIqV92AaeXatLxBI9gBaebbnrfifHhDYfgasaacH8akY=wiFfYdH8Gipec8Eeeu0xXdbba9frFj0=OqFfea0dXdd9vqai=hGuQ8kuc9pgc9s8qqaq=dirpe0xb9q8qiLsFr0=vr0=vr0dc8meaabaqaciaacaGaaeqabaqabeGadaaakeaacqWGqbaudaWgaaWcbaGaemOAaOMaeiikaGIaemyAaKMaeiykaKcabeaakiabc6ha+jabd6eaojabcIcaOiabicdaWiabcYcaSGGaciab=n8aZnaaDaaaleaacqWGqbaucqGGSaalcqWGPbqAaeaacqaIYaGmaaGccqGGPaqkaaa@3EF5@, with σP,i2=τi2T=Jτi2N
 MathType@MTEF@5@5@+=feaafiart1ev1aaatCvAUfKttLearuWrP9MDH5MBPbIqV92AaeXatLxBI9gBaebbnrfifHhDYfgasaacH8akY=wiFfYdH8Gipec8Eeeu0xXdbba9frFj0=OqFfea0dXdd9vqai=hGuQ8kuc9pgc9s8qqaq=dirpe0xb9q8qiLsFr0=vr0=vr0dc8meaabaqaciaacaGaaeqabaqabeGadaaakeaaiiGacqWFdpWCdaqhaaWcbaGaemiuaaLaeiilaWIaemyAaKgabaGaeGOmaidaaOGaeyypa0ZaaSaaaeaacqWFepaDdaqhaaWcbaGaemyAaKgabaGaeGOmaidaaaGcbaGaemivaqfaaiabg2da9maalaaabaGaemOsaOKae8hXdq3aa0baaSqaaiabdMgaPbqaaiabikdaYaaaaOqaaiabd6eaobaaaaa@412A@. Finally, {*E*_*ijk*_} are independent *N*(0,1) random variables incorporating the additional variability due to the measurement process. See below for more details regarding the specification σP,i2=τi2T
 MathType@MTEF@5@5@+=feaafiart1ev1aaatCvAUfKttLearuWrP9MDH5MBPbIqV92AaeXatLxBI9gBaebbnrfifHhDYfgasaacH8akY=wiFfYdH8Gipec8Eeeu0xXdbba9frFj0=OqFfea0dXdd9vqai=hGuQ8kuc9pgc9s8qqaq=dirpe0xb9q8qiLsFr0=vr0=vr0dc8meaabaqaciaacaGaaeqabaqabeGadaaakeaaiiGacqWFdpWCdaqhaaWcbaGaemiuaaLaeiilaWIaemyAaKgabaGaeGOmaidaaOGaeyypa0ZaaSaaaeaacqWFepaDdaqhaaWcbaGaemyAaKgabaGaeGOmaidaaaGcbaGaemivaqfaaaaa@398E@. It follows that

var⁡(Yijk)=σP,i2+σE2=τi2T+σE2=Jτi2N+σE2.
 MathType@MTEF@5@5@+=feaafiart1ev1aaatCvAUfKttLearuWrP9MDH5MBPbIqV92AaeXatLxBI9gBaebbnrfifHhDYfgasaacH8akY=wiFfYdH8Gipec8Eeeu0xXdbba9frFj0=OqFfea0dXdd9vqai=hGuQ8kuc9pgc9s8qqaq=dirpe0xb9q8qiLsFr0=vr0=vr0dc8meaabaqaciaacaGaaeqabaqabeGadaaakeaacyGG2bGDcqGGHbqycqGGYbGCcqGGOaakcqWGzbqwdaWgaaWcbaGaemyAaKMaemOAaOMaem4AaSgabeaakiabcMcaPiabg2da9GGaciab=n8aZnaaDaaaleaacqWGqbaucqGGSaalcqWGPbqAaeaacqaIYaGmaaGccqGHRaWkcqWFdpWCdaqhaaWcbaGaemyraueabaGaeGOmaidaaOGaeyypa0ZaaSaaaeaacqWFepaDdaqhaaWcbaGaemyAaKgabaGaeGOmaidaaaGcbaGaemivaqfaaiabgUcaRiab=n8aZnaaDaaaleaacqWGfbqraeaacqaIYaGmaaGccqGH9aqpdaWcaaqaaiabdQeakjab=r8a0naaDaaaleaacqWGPbqAaeaacqaIYaGmaaaakeaacqWGobGtaaGaey4kaSIae83Wdm3aa0baaSqaaiabdweafbqaaiabikdaYaaakiabc6caUaaa@5D0E@

Here, var(*Y*_*ijk*_) is modeled as the sum of two components of variance. The first, σP,i2=Jτi2N
 MathType@MTEF@5@5@+=feaafiart1ev1aaatCvAUfKttLearuWrP9MDH5MBPbIqV92AaeXatLxBI9gBaebbnrfifHhDYfgasaacH8akY=wiFfYdH8Gipec8Eeeu0xXdbba9frFj0=OqFfea0dXdd9vqai=hGuQ8kuc9pgc9s8qqaq=dirpe0xb9q8qiLsFr0=vr0=vr0dc8meaabaqaciaacaGaaeqabaqabeGadaaakeaaiiGacqWFdpWCdaqhaaWcbaGaemiuaaLaeiilaWIaemyAaKgabaGaeGOmaidaaOGaeyypa0ZaaSaaaeaacqWGkbGscqWFepaDdaqhaaWcbaGaemyAaKgabaGaeGOmaidaaaGcbaGaemOta4eaaaaa@3A9F@, is due to the sampling variation of the frequency of allele 2 in the subjects assigned to each pool. The second, σE2
 MathType@MTEF@5@5@+=feaafiart1ev1aaatCvAUfKttLearuWrP9MDH5MBPbIqV92AaeXatLxBI9gBaebbnrfifHhDYfgasaacH8akY=wiFfYdH8Gipec8Eeeu0xXdbba9frFj0=OqFfea0dXdd9vqai=hGuQ8kuc9pgc9s8qqaq=dirpe0xb9q8qiLsFr0=vr0=vr0dc8meaabaqaciaacaGaaeqabaqabeGadaaakeaaiiGacqWFdpWCdaqhaaWcbaGaemyraueabaGaeGOmaidaaaaa@30A8@, is due to the measurement error of the processing of the pooled material. Under an ideal measurement process, σE2
 MathType@MTEF@5@5@+=feaafiart1ev1aaatCvAUfKttLearuWrP9MDH5MBPbIqV92AaeXatLxBI9gBaebbnrfifHhDYfgasaacH8akY=wiFfYdH8Gipec8Eeeu0xXdbba9frFj0=OqFfea0dXdd9vqai=hGuQ8kuc9pgc9s8qqaq=dirpe0xb9q8qiLsFr0=vr0=vr0dc8meaabaqaciaacaGaaeqabaqabeGadaaakeaaiiGacqWFdpWCdaqhaaWcbaGaemyraueabaGaeGOmaidaaaaa@30A8@ = 0; we define a parameter *m *to capture the departure from this ideal. The parameter *m *(machine replicability variance factor), *m *≥ 1, is defined by σE2=σP,i2(m−1)
 MathType@MTEF@5@5@+=feaafiart1ev1aaatCvAUfKttLearuWrP9MDH5MBPbIqV92AaeXatLxBI9gBaebbnrfifHhDYfgasaacH8akY=wiFfYdH8Gipec8Eeeu0xXdbba9frFj0=OqFfea0dXdd9vqai=hGuQ8kuc9pgc9s8qqaq=dirpe0xb9q8qiLsFr0=vr0=vr0dc8meaabaqaciaacaGaaeqabaqabeGadaaakeaaiiGacqWFdpWCdaqhaaWcbaGaemyraueabaGaeGOmaidaaOGaeyypa0Jae83Wdm3aa0baaSqaaiabdcfaqjabcYcaSiabdMgaPbqaaiabikdaYaaakiabcIcaOiabd2gaTjabgkHiTiabigdaXiabcMcaPaaa@3CF5@, so that *m *= 1 represents the ideal measurement process and *m *> 1 models additional variability due to a less than perfect measurement process. The fraction of var(*Y*_*ijk*_) due to the measurement process is m−1m
 MathType@MTEF@5@5@+=feaafiart1ev1aaatCvAUfKttLearuWrP9MDH5MBPbIqV92AaeXatLxBI9gBaebbnrfifHhDYfgasaacH8akY=wiFfYdH8Gipec8Eeeu0xXdbba9frFj0=OqFfea0dXdd9vqai=hGuQ8kuc9pgc9s8qqaq=dirpe0xb9q8qiLsFr0=vr0=vr0dc8meaabaqaciaacaGaaeqabaqabeGadaaakeaadaWcaaqaaiabd2gaTjabgkHiTiabigdaXaqaaiabd2gaTbaaaaa@315F@.

This model is dependent on the assumption that E(Πi)=E(AijkAijk+Bijk)=E(Yijk)
 MathType@MTEF@5@5@+=feaafiart1ev1aaatCvAUfKttLearuWrP9MDH5MBPbIqV92AaeXatLxBI9gBaebbnrfifHhDYfgasaacH8akY=wiFfYdH8Gipec8Eeeu0xXdbba9frFj0=OqFfea0dXdd9vqai=hGuQ8kuc9pgc9s8qqaq=dirpe0xb9q8qiLsFr0=vr0=vr0dc8meaabaqaciaacaGaaeqabaqabeGadaaakeaacqWGfbqrcqGGOaakcqqHGoaudaWgaaWcbaGaemyAaKgabeaakiabcMcaPiabg2da9iabdweafjabcIcaOmaalaaabaGaemyqae0aaSbaaSqaaiabdMgaPjabdQgaQjabdUgaRbqabaaakeaacqWGbbqqdaWgaaWcbaGaemyAaKMaemOAaOMaem4AaSgabeaakiabgUcaRiabdkeacnaaBaaaleaacqWGPbqAcqWGQbGAcqWGRbWAaeqaaaaakiabcMcaPiabg2da9iabdweafjabcIcaOiabdMfaznaaBaaaleaacqWGPbqAcqWGQbGAcqWGRbWAaeqaaOGaeiykaKcaaa@509A@. Also, let ρ=max⁡(σP,02,σP,12)min⁡(σP,02,σP,12)
 MathType@MTEF@5@5@+=feaafiart1ev1aaatCvAUfKttLearuWrP9MDH5MBPbIqV92AaeXatLxBI9gBaebbnrfifHhDYfgasaacH8akY=wiFfYdH8Gipec8Eeeu0xXdbba9frFj0=OqFfea0dXdd9vqai=hGuQ8kuc9pgc9s8qqaq=dirpe0xb9q8qiLsFr0=vr0=vr0dc8meaabaqaciaacaGaaeqabaqabeGadaaakeaaiiGacqWFbpGCcqGH9aqpdaWcaaqaaiGbc2gaTjabcggaHjabcIha4jabcIcaOiab=n8aZnaaDaaaleaacqWGqbaucqGGSaalcqaIWaamaeaacqaIYaGmaaGccqGGSaalcqWFdpWCdaqhaaWcbaGaemiuaaLaeiilaWIaeGymaedabaGaeGOmaidaaOGaeiykaKcabaGagiyBa0MaeiyAaKMaeiOBa4MaeiikaGIae83Wdm3aa0baaSqaaiabdcfaqjabcYcaSiabicdaWaqaaiabikdaYaaakiabcYcaSiab=n8aZnaaDaaaleaacqWGqbaucqGGSaalcqaIXaqmaeaacqaIYaGmaaGccqGGPaqkaaaaaa@5471@. This value is an indication of the adequacy of the approximation of the NCP in equation (1) below [28].

Let

Yij•=∑k=1KYijkK,Yi••=∑j=1J∑k=1KYijkJK, and Y•••=∑i=01∑j=1J∑k=1KYijk2JK.
 MathType@MTEF@5@5@+=feaafiart1ev1aaatCvAUfKttLearuWrP9MDH5MBPbIqV92AaeXatLxBI9gBaebbnrfifHhDYfgasaacH8akY=wiFfYdH8Gipec8Eeeu0xXdbba9frFj0=OqFfea0dXdd9vqai=hGuQ8kuc9pgc9s8qqaq=dirpe0xb9q8qiLsFr0=vr0=vr0dc8meaabaqaciaacaGaaeqabaqabeGadaaakeaacqWGzbqwdaWgaaWcbaGaemyAaKMaemOAaOMaeyOiGClabeaakiabg2da9maalaaabaWaaabCaeaacqWGzbqwdaWgaaWcbaGaemyAaKMaemOAaOMaem4AaSgabeaaaeaacqWGRbWAcqGH9aqpcqaIXaqmaeaacqWGlbWsa0GaeyyeIuoaaOqaaiabdUealbaacqGGSaalcqWGzbqwdaWgaaWcbaGaemyAaKMaeyOiGCRaeyOiGClabeaakiabg2da9maalaaabaWaaabCaeaadaaeWbqaaiabdMfaznaaBaaaleaacqWGPbqAcqWGQbGAcqWGRbWAaeqaaaqaaiabdUgaRjabg2da9iabigdaXaqaaiabdUealbqdcqGHris5aaWcbaGaemOAaOMaeyypa0JaeGymaedabaGaemOsaOeaniabggHiLdaakeaacqWGkbGscqWGlbWsaaGaeiilaWIaeeiiaaIaeeyyaeMaeeOBa4MaeeizaqMaeeiiaaIaemywaK1aaSbaaSqaaiabgkci3kabgkci3kabgkci3cqabaGccqGH9aqpdaWcaaqaamaaqahabaWaaabCaeaadaaeWbqaaiabdMfaznaaBaaaleaacqWGPbqAcqWGQbGAcqWGRbWAaeqaaaqaaiabdUgaRjabg2da9iabigdaXaqaaiabdUealbqdcqGHris5aaWcbaGaemOAaOMaeyypa0JaeGymaedabaGaemOsaOeaniabggHiLdaaleaacqWGPbqAcqGH9aqpcqaIWaamaeaacqaIXaqma0GaeyyeIuoaaOqaaiabikdaYiabdQeakjabdUealbaacqGGUaGlaaa@88A6@

Following Scheffé [29], the means used in the sums of squares can be expressed in terms of the ANOVA model as

Yij•=∑k=1KYijkK=μ+αi+Pj(i)+σEEij•, where Eij•=∑k=1KEijk/K;
 MathType@MTEF@5@5@+=feaafiart1ev1aaatCvAUfKttLearuWrP9MDH5MBPbIqV92AaeXatLxBI9gBaebbnrfifHhDYfgasaacH8akY=wiFfYdH8Gipec8Eeeu0xXdbba9frFj0=OqFfea0dXdd9vqai=hGuQ8kuc9pgc9s8qqaq=dirpe0xb9q8qiLsFr0=vr0=vr0dc8meaabaqaciaacaGaaeqabaqabeGadaaakeaacqWGzbqwdaWgaaWcbaGaemyAaKMaemOAaOMaeyOiGClabeaakiabg2da9maalaaabaWaaabCaeaacqWGzbqwdaWgaaWcbaGaemyAaKMaemOAaOMaem4AaSgabeaaaeaacqWGRbWAcqGH9aqpcqaIXaqmaeaacqWGlbWsa0GaeyyeIuoaaOqaaiabdUealbaacqGH9aqpiiGacqWF8oqBcqGHRaWkcqWFXoqydaWgaaWcbaGaemyAaKgabeaakiabgUcaRiabdcfaqnaaBaaaleaacqWGQbGAcqGGOaakcqWGPbqAcqGGPaqkaeqaaOGaey4kaSIae83Wdm3aaSbaaSqaaiabdweafbqabaGccqWGfbqrdaWgaaWcbaGaemyAaKMaemOAaOMaeyOiGClabeaakiabcYcaSiabbccaGiabbEha3jabbIgaOjabbwgaLjabbkhaYjabbwgaLjabbccaGiabdweafnaaBaaaleaacqWGPbqAcqWGQbGAcqGHIaYTaeqaaOGaeyypa0ZaaabCaeaacqWGfbqrdaWgaaWcbaGaemyAaKMaemOAaOMaem4AaSgabeaaaeaacqWGRbWAcqGH9aqpcqaIXaqmaeaacqWGlbWsa0GaeyyeIuoakiabc+caViabdUealjabcUda7aaa@768D@

Yi••=∑j=1J∑k=1KYijkJK=μ+αi+P•(i)+σEEi••, where P•(i)=∑j=1JPj(i)/J and Ei••=∑j=1J∑k=1KEijk/JK;
 MathType@MTEF@5@5@+=feaafiart1ev1aaatCvAUfKttLearuWrP9MDH5MBPbIqV92AaeXatLxBI9gBaebbnrfifHhDYfgasaacH8akY=wiFfYdH8Gipec8Eeeu0xXdbba9frFj0=OqFfea0dXdd9vqai=hGuQ8kuc9pgc9s8qqaq=dirpe0xb9q8qiLsFr0=vr0=vr0dc8meaabaqaciaacaGaaeqabaqabeGadaaakeaacqWGzbqwdaWgaaWcbaGaemyAaKMaeyOiGCRaeyOiGClabeaakiabg2da9maalaaabaWaaabCaeaadaaeWbqaaiabdMfaznaaBaaaleaacqWGPbqAcqWGQbGAcqWGRbWAaeqaaaqaaiabdUgaRjabg2da9iabigdaXaqaaiabdUealbqdcqGHris5aaWcbaGaemOAaOMaeyypa0JaeGymaedabaGaemOsaOeaniabggHiLdaakeaacqWGkbGscqWGlbWsaaGaeyypa0dcciGae8hVd0Maey4kaSIae8xSde2aaSbaaSqaaiabdMgaPbqabaGccqGHRaWkcqWGqbaudaWgaaWcbaGaeyOiGCRaeiikaGIaemyAaKMaeiykaKcabeaakiabgUcaRiab=n8aZnaaBaaaleaacqWGfbqraeqaaOGaemyrau0aaSbaaSqaaiabdMgaPjabgkci3kabgkci3cqabaGccqGGSaalcqqGGaaicqqG3bWDcqqGObaAcqqGLbqzcqqGYbGCcqqGLbqzcqqGGaaicqWGqbaudaWgaaWcbaGaeyOiGCRaeiikaGIaemyAaKMaeiykaKcabeaakiabg2da9maaqahabaGaemiuaa1aaSbaaSqaaiabdQgaQjabcIcaOiabdMgaPjabcMcaPaqabaaabaGaemOAaOMaeyypa0JaeGymaedabaGaemOsaOeaniabggHiLdGccqGGVaWlcqWGkbGscqqGGaaicqqGHbqycqqGUbGBcqqGKbazcqqGGaaicqWGfbqrdaWgaaWcbaGaemyAaKMaeyOiGCRaeyOiGClabeaakiabg2da9maaqahabaWaaabCaeaacqWGfbqrdaWgaaWcbaGaemyAaKMaemOAaOMaem4AaSgabeaaaeaacqWGRbWAcqGH9aqpcqaIXaqmaeaacqWGlbWsa0GaeyyeIuoakiabc+caViabdQeakjabdUealbWcbaGaemOAaOMaeyypa0JaeGymaedabaGaemOsaOeaniabggHiLdGccqGG7aWoaaa@A1C8@

Y•••=∑i=01∑j=1J∑k=1KYijk2JK=μ+0+P•(•)+σEE•••, where P•(•)=∑i=01P•(i)/2 and E•••=∑i=01Ei••/2.
 MathType@MTEF@5@5@+=feaafiart1ev1aaatCvAUfKttLearuWrP9MDH5MBPbIqV92AaeXatLxBI9gBaebbnrfifHhDYfgasaacH8akY=wiFfYdH8Gipec8Eeeu0xXdbba9frFj0=OqFfea0dXdd9vqai=hGuQ8kuc9pgc9s8qqaq=dirpe0xb9q8qiLsFr0=vr0=vr0dc8meaabaqaciaacaGaaeqabaqabeGadaaakeaacqWGzbqwdaWgaaWcbaGaeyOiGCRaeyOiGCRaeyOiGClabeaakiabg2da9maalaaabaWaaabCaeaadaaeWbqaamaaqahabaGaemywaK1aaSbaaSqaaiabdMgaPjabdQgaQjabdUgaRbqabaaabaGaem4AaSMaeyypa0JaeGymaedabaGaem4saSeaniabggHiLdaaleaacqWGQbGAcqGH9aqpcqaIXaqmaeaacqWGkbGsa0GaeyyeIuoaaSqaaiabdMgaPjabg2da9iabicdaWaqaaiabigdaXaqdcqGHris5aaGcbaGaeGOmaiJaemOsaOKaem4saSeaaiabg2da9GGaciab=X7aTjabgUcaRiabicdaWiabgUcaRiabdcfaqnaaBaaaleaacqGHIaYTcqGGOaakcqGHIaYTcqGGPaqkaeqaaOGaey4kaSIae83Wdm3aaSbaaSqaaiabdweafbqabaGccqWGfbqrdaWgaaWcbaGaeyOiGCRaeyOiGCRaeyOiGClabeaakiabcYcaSiabbccaGiabbEha3jabbIgaOjabbwgaLjabbkhaYjabbwgaLjabbccaGiabdcfaqnaaBaaaleaacqGHIaYTcqGGOaakcqGHIaYTcqGGPaqkaeqaaOGaeyypa0ZaaabCaeaacqWGqbaudaWgaaWcbaGaeyOiGCRaeiikaGIaemyAaKMaeiykaKcabeaaaeaacqWGPbqAcqGH9aqpcqaIWaamaeaacqaIXaqma0GaeyyeIuoakiabc+caViabikdaYiabbccaGiabbggaHjabb6gaUjabbsgaKjabbccaGiabdweafnaaBaaaleaacqGHIaYTcqGHIaYTcqGHIaYTaeqaaOGaeyypa0ZaaabCaeaacqWGfbqrdaWgaaWcbaGaemyAaKMaeyOiGCRaeyOiGClabeaaaeaacqWGPbqAcqGH9aqpcqaIWaamaeaacqaIXaqma0GaeyyeIuoakiabc+caViabikdaYiabc6caUaaa@9F95@

Then,

Yij•−Yi••=Pj(i)+σEEij•−(P•(i)+σEEi••)=Tij−Ti•,
 MathType@MTEF@5@5@+=feaafiart1ev1aaatCvAUfKttLearuWrP9MDH5MBPbIqV92AaeXatLxBI9gBaebbnrfifHhDYfgasaacH8akY=wiFfYdH8Gipec8Eeeu0xXdbba9frFj0=OqFfea0dXdd9vqai=hGuQ8kuc9pgc9s8qqaq=dirpe0xb9q8qiLsFr0=vr0=vr0dc8meaabaqaciaacaGaaeqabaqabeGadaaakeaacqWGzbqwdaWgaaWcbaGaemyAaKMaemOAaOMaeyOiGClabeaakiabgkHiTiabdMfaznaaBaaaleaacqWGPbqAcqGHIaYTcqGHIaYTaeqaaOGaeyypa0Jaemiuaa1aaSbaaSqaaiabdQgaQjabcIcaOiabdMgaPjabcMcaPaqabaGccqGHRaWkiiGacqWFdpWCdaWgaaWcbaGaemyraueabeaakiabdweafnaaBaaaleaacqWGPbqAcqWGQbGAcqGHIaYTaeqaaOGaeyOeI0IaeiikaGIaemiuaa1aaSbaaSqaaiabgkci3kabcIcaOiabdMgaPjabcMcaPaqabaGccqGHRaWkcqWFdpWCdaWgaaWcbaGaemyraueabeaakiabdweafnaaBaaaleaacqWGPbqAcqGHIaYTcqGHIaYTaeqaaOGaeiykaKIaeyypa0Jaemivaq1aaSbaaSqaaiabdMgaPjabdQgaQbqabaGccqGHsislcqWGubavdaWgaaWcbaGaemyAaKMaeyOiGClabeaakiabcYcaSaaa@66C7@

where Tij=Pj(i)+σEEij•,Ti•=∑j=1JTij/J, and Tij~N(0,σP,i2+σE2K)
 MathType@MTEF@5@5@+=feaafiart1ev1aaatCvAUfKttLearuWrP9MDH5MBPbIqV92AaeXatLxBI9gBaebbnrfifHhDYfgasaacH8akY=wiFfYdH8Gipec8Eeeu0xXdbba9frFj0=OqFfea0dXdd9vqai=hGuQ8kuc9pgc9s8qqaq=dirpe0xb9q8qiLsFr0=vr0=vr0dc8meaabaqaciaacaGaaeqabaqabeGadaaakeaacqWGubavdaWgaaWcbaGaemyAaKMaemOAaOgabeaakiabg2da9iabdcfaqnaaBaaaleaacqWGQbGAcqGGOaakcqWGPbqAcqGGPaqkaeqaaOGaey4kaSccciGae83Wdm3aaSbaaSqaaiabdweafbqabaGccqWGfbqrdaWgaaWcbaGaemyAaKMaemOAaOMaeyOiGClabeaakiabcYcaSiabdsfaunaaBaaaleaacqWGPbqAcqGHIaYTaeqaaOGaeyypa0ZaaabCaeaacqWGubavdaWgaaWcbaGaemyAaKMaemOAaOgabeaaaeaacqWGQbGAcqGH9aqpcqaIXaqmaeaacqWGkbGsa0GaeyyeIuoakiabc+caViabdQeakjabcYcaSiabbccaGiabbggaHjabb6gaUjabbsgaKjabbccaGiabdsfaunaaBaaaleaacqWGPbqAcqWGQbGAaeqaaOGaeiOFa4NaemOta4KaeiikaGIaeGimaaJaeiilaWIae83Wdm3aa0baaSqaaiabdcfaqjabcYcaSiabdMgaPbqaaiabikdaYaaakiabgUcaRmaalaaabaGae83Wdm3aa0baaSqaaiabdweafbqaaiabikdaYaaaaOqaaiabdUealbaacqGGPaqkaaa@7112@.

If we let *W*_*i *_represent *n *independent and identically distributed *N*(*μ*, *σ*^2^) random variables, then ∑i=1n(Wi−W¯)2
 MathType@MTEF@5@5@+=feaafiart1ev1aaatCvAUfKttLearuWrP9MDH5MBPbIqV92AaeXatLxBI9gBaebbnrfifHhDYfgasaacH8akY=wiFfYdH8Gipec8Eeeu0xXdbba9frFj0=OqFfea0dXdd9vqai=hGuQ8kuc9pgc9s8qqaq=dirpe0xb9q8qiLsFr0=vr0=vr0dc8meaabaqaciaacaGaaeqabaqabeGadaaakeaadaaeWbqaaiabcIcaOiabdEfaxnaaBaaaleaacqWGPbqAaeqaaOGaeyOeI0Iafm4vaCLbaebacqGGPaqkdaahaaWcbeqaaiabikdaYaaaaeaacqWGPbqAcqGH9aqpcqaIXaqmaeaacqWGUbGBa0GaeyyeIuoaaaa@3B6E@ has the distribution σ2χn−12
 MathType@MTEF@5@5@+=feaafiart1ev1aaatCvAUfKttLearuWrP9MDH5MBPbIqV92AaeXatLxBI9gBaebbnrfifHhDYfgasaacH8akY=wiFfYdH8Gipec8Eeeu0xXdbba9frFj0=OqFfea0dXdd9vqai=hGuQ8kuc9pgc9s8qqaq=dirpe0xb9q8qiLsFr0=vr0=vr0dc8meaabaqaciaacaGaaeqabaqabeGadaaakeaaiiGacqWFdpWCdaahaaWcbeqaaiabikdaYaaakiab=D8aJnaaDaaaleaacqWGUbGBcqGHsislcqaIXaqmaeaacqaIYaGmaaaaaa@35B2@[18]. Consequently,

SSP=K∑i∑j(Yij•−Yi••)2=K∑i[∑j(Tij−Ti•)2]=K∑i(σP,i2+σE2K)Xi,
 MathType@MTEF@5@5@+=feaafiart1ev1aaatCvAUfKttLearuWrP9MDH5MBPbIqV92AaeXatLxBI9gBaebbnrfifHhDYfgasaacH8akY=wiFfYdH8Gipec8Eeeu0xXdbba9frFj0=OqFfea0dXdd9vqai=hGuQ8kuc9pgc9s8qqaq=dirpe0xb9q8qiLsFr0=vr0=vr0dc8meaabaqaciaacaGaaeqabaqabeGadaaakqaaeeqaaiabdofatjabdofatnaaBaaaleaacqWGqbauaeqaaOGaeyypa0Jaem4saS0aaabuaeaadaaeqbqaaiabcIcaOiabdMfaznaaBaaaleaacqWGPbqAcqWGQbGAcqGHIaYTaeqaaOGaeyOeI0caleaacqWGQbGAaeqaniabggHiLdGccqWGzbqwdaWgaaWcbaGaemyAaKMaeyOiGCRaeyOiGClabeaakiabcMcaPmaaCaaaleqabaGaeGOmaidaaaqaaiabdMgaPbqab0GaeyyeIuoaaOqaaiabg2da9iabdUealnaaqafabaGaei4waS1aaabuaeaacqGGOaakcqWGubavdaWgaaWcbaGaemyAaKMaemOAaOgabeaakiabgkHiTaWcbaGaemOAaOgabeqdcqGHris5aOGaemivaq1aaSbaaSqaaiabdMgaPjabgkci3cqabaGccqGGPaqkdaahaaWcbeqaaiabikdaYaaaaeaacqWGPbqAaeqaniabggHiLdGccqGGDbqxaeaacqGH9aqpcqWGlbWsdaaeqbqaaiabcIcaOGGaciab=n8aZnaaDaaaleaacqWGqbaucqGGSaalcqWGPbqAaeaacqaIYaGmaaGccqGHRaWkdaWcaaqaaiab=n8aZnaaDaaaleaacqWGfbqraeaacqaIYaGmaaaakeaacqWGlbWsaaGaeiykaKcaleaacqWGPbqAaeqaniabggHiLdGccqWGybawdaWgaaWcbaGaemyAaKgabeaakiabcYcaSaaaaa@781D@

where Xi~χJ−12
 MathType@MTEF@5@5@+=feaafiart1ev1aaatCvAUfKttLearuWrP9MDH5MBPbIqV92AaeXatLxBI9gBaebbnrfifHhDYfgasaacH8akY=wiFfYdH8Gipec8Eeeu0xXdbba9frFj0=OqFfea0dXdd9vqai=hGuQ8kuc9pgc9s8qqaq=dirpe0xb9q8qiLsFr0=vr0=vr0dc8meaabaqaciaacaGaaeqabaqabeGadaaakeaacqWGybawdaWgaaWcbaGaemyAaKgabeaakiabc6ha+HGaciab=D8aJnaaDaaaleaacqWGkbGscqGHsislcqaIXaqmaeaacqaIYaGmaaaaaa@36D1@. The sum of squares *SS*_*P *_therefore has the distribution K(σP¯2+σE2K)χ2(J−1)2
 MathType@MTEF@5@5@+=feaafiart1ev1aaatCvAUfKttLearuWrP9MDH5MBPbIqV92AaeXatLxBI9gBaebbnrfifHhDYfgasaacH8akY=wiFfYdH8Gipec8Eeeu0xXdbba9frFj0=OqFfea0dXdd9vqai=hGuQ8kuc9pgc9s8qqaq=dirpe0xb9q8qiLsFr0=vr0=vr0dc8meaabaqaciaacaGaaeqabaqabeGadaaakeaacqWGlbWscqGGOaakiiGacqWFdpWCdaqhaaWcbaGafmiuaaLbaebaaeaacqaIYaGmaaGccqGHRaWkdaWcaaqaaiab=n8aZnaaDaaaleaacqWGfbqraeaacqaIYaGmaaaakeaacqWGlbWsaaGaeiykaKIae83Xdm2aa0baaSqaaiabikdaYiabcIcaOiabdQeakjabgkHiTiabigdaXiabcMcaPaqaaiabikdaYaaaaaa@422B@ when the null hypothesis is true, with σP¯2=σP,02=σP,12
 MathType@MTEF@5@5@+=feaafiart1ev1aaatCvAUfKttLearuWrP9MDH5MBPbIqV92AaeXatLxBI9gBaebbnrfifHhDYfgasaacH8akY=wiFfYdH8Gipec8Eeeu0xXdbba9frFj0=OqFfea0dXdd9vqai=hGuQ8kuc9pgc9s8qqaq=dirpe0xb9q8qiLsFr0=vr0=vr0dc8meaabaqaciaacaGaaeqabaqabeGadaaakeaaiiGacqWFdpWCdaqhaaWcbaGafmiuaaLbaebaaeaacqaIYaGmaaGccqGH9aqpcqWFdpWCdaqhaaWcbaGaemiuaaLaeiilaWIaeGimaadabaGaeGOmaidaaOGaeyypa0Jae83Wdm3aa0baaSqaaiabdcfaqjabcYcaSiabigdaXaqaaiabikdaYaaaaaa@3EA0@. Further E(SSP)=2(J−1)(KσP¯2+σE2)
 MathType@MTEF@5@5@+=feaafiart1ev1aaatCvAUfKttLearuWrP9MDH5MBPbIqV92AaeXatLxBI9gBaebbnrfifHhDYfgasaacH8akY=wiFfYdH8Gipec8Eeeu0xXdbba9frFj0=OqFfea0dXdd9vqai=hGuQ8kuc9pgc9s8qqaq=dirpe0xb9q8qiLsFr0=vr0=vr0dc8meaabaqaciaacaGaaeqabaqabeGadaaakeaacqWGfbqrcqGGOaakcqWGtbWucqWGtbWudaWgaaWcbaGaemiuaafabeaakiabcMcaPiabg2da9iabikdaYiabcIcaOiabdQeakjabgkHiTiabigdaXiabcMcaPiabcIcaOiabdUealHGaciab=n8aZnaaDaaaleaacuWGqbaugaqeaaqaaiabikdaYaaakiabgUcaRiab=n8aZnaaDaaaleaacqWGfbqraeaacqaIYaGmaaGccqGGPaqkaaa@45B3@ under both the null and alternative hypotheses with σP¯2=Jτ¯2N
 MathType@MTEF@5@5@+=feaafiart1ev1aaatCvAUfKttLearuWrP9MDH5MBPbIqV92AaeXatLxBI9gBaebbnrfifHhDYfgasaacH8akY=wiFfYdH8Gipec8Eeeu0xXdbba9frFj0=OqFfea0dXdd9vqai=hGuQ8kuc9pgc9s8qqaq=dirpe0xb9q8qiLsFr0=vr0=vr0dc8meaabaqaciaacaGaaeqabaqabeGadaaakeaaiiGacqWFdpWCdaqhaaWcbaGafmiuaaLbaebaaeaacqaIYaGmaaGccqGH9aqpdaWcaaqaaiabdQeakjqb=r8a0zaaraWaaWbaaSqabeaacqaIYaGmaaaakeaacqWGobGtaaaaaa@3739@ and τ¯2=∑i=01τi22=∑var⁡(Πi)2
 MathType@MTEF@5@5@+=feaafiart1ev1aaatCvAUfKttLearuWrP9MDH5MBPbIqV92AaeXatLxBI9gBaebbnrfifHhDYfgasaacH8akY=wiFfYdH8Gipec8Eeeu0xXdbba9frFj0=OqFfea0dXdd9vqai=hGuQ8kuc9pgc9s8qqaq=dirpe0xb9q8qiLsFr0=vr0=vr0dc8meaabaqaciaacaGaaeqabaqabeGadaaakeaaiiGacuWFepaDgaqeamaaCaaaleqabaGaeGOmaidaaOGaeyypa0ZaaSaaaeaadaaeWbqaaiab=r8a0naaDaaaleaacqWGPbqAaeaacqaIYaGmaaaabaGaemyAaKMaeyypa0JaeGimaadabaGaeGymaedaniabggHiLdaakeaacqaIYaGmaaGaeyypa0ZaaSaaaeaadaaeabqaaiGbcAha2jabcggaHjabckhaYjabcIcaOiabfc6aqnaaBaaaleaacqWGPbqAaeqaaOGaeiykaKcaleqabeqdcqGHris5aaGcbaGaeGOmaidaaaaa@498E@. The distribution of *SS*_*P *_under the alternative is a weighted sum of independent central chi-square distributions.

To obtain the distribution of *SS*_*A*_, consider

Yi••−Y•••=(αi+P•(i)+σEEi••)−(P•(•)+σEE•••)=αi+Si−S•,
 MathType@MTEF@5@5@+=feaafiart1ev1aaatCvAUfKttLearuWrP9MDH5MBPbIqV92AaeXatLxBI9gBaebbnrfifHhDYfgasaacH8akY=wiFfYdH8Gipec8Eeeu0xXdbba9frFj0=OqFfea0dXdd9vqai=hGuQ8kuc9pgc9s8qqaq=dirpe0xb9q8qiLsFr0=vr0=vr0dc8meaabaqaciaacaGaaeqabaqabeGadaaakeaacqWGzbqwdaWgaaWcbaGaemyAaKMaeyOiGCRaeyOiGClabeaakiabgkHiTiabdMfaznaaBaaaleaacqGHIaYTcqGHIaYTcqGHIaYTaeqaaOGaeyypa0JaeiikaGccciGae8xSde2aaSbaaSqaaiabdMgaPbqabaGccqGHRaWkcqWGqbaudaWgaaWcbaGaeyOiGCRaeiikaGIaemyAaKMaeiykaKcabeaakiabgUcaRiab=n8aZnaaBaaaleaacqWGfbqraeqaaOGaemyrau0aaSbaaSqaaiabdMgaPjabgkci3kabgkci3cqabaGccqGGPaqkcqGHsislcqGGOaakcqWGqbaudaWgaaWcbaGaeyOiGCRaeiikaGIaeyOiGCRaeiykaKcabeaakiabgUcaRiab=n8aZnaaBaaaleaacqWGfbqraeqaaOGaemyrau0aaSbaaSqaaiabgkci3kabgkci3kabgkci3cqabaGccqGGPaqkcqGH9aqpcqWFXoqydaWgaaWcbaGaemyAaKgabeaakiabgUcaRiabdofatnaaBaaaleaacqWGPbqAaeqaaOGaeyOeI0Iaem4uam1aaSbaaSqaaiabgkci3cqabaGccqGGSaalaaa@6ECD@

where Si=P•(i)+σEEi•• with Si~N(0,σP,i2J+σE2JK)
 MathType@MTEF@5@5@+=feaafiart1ev1aaatCvAUfKttLearuWrP9MDH5MBPbIqV92AaeXatLxBI9gBaebbnrfifHhDYfgasaacH8akY=wiFfYdH8Gipec8Eeeu0xXdbba9frFj0=OqFfea0dXdd9vqai=hGuQ8kuc9pgc9s8qqaq=dirpe0xb9q8qiLsFr0=vr0=vr0dc8meaabaqaciaacaGaaeqabaqabeGadaaakeaacqWGtbWudaWgaaWcbaGaemyAaKgabeaakiabg2da9iabdcfaqnaaBaaaleaacqGHIaYTcqGGOaakcqWGPbqAcqGGPaqkaeqaaOGaey4kaSccciGae83Wdm3aaSbaaSqaaiabdweafbqabaGccqWGfbqrdaWgaaWcbaGaemyAaKMaeyOiGCRaeyOiGClabeaakiabbccaGiabbEha3jabbMgaPjabbsha0jabbIgaOjabbccaGiabdofatnaaBaaaleaacqWGPbqAaeqaaOGaeiOFa4NaemOta4KaeiikaGIaeGimaaJaeiilaWYaaSaaaeaacqWFdpWCdaqhaaWcbaGaemiuaaLaeiilaWIaemyAaKgabaGaeGOmaidaaaGcbaGaemOsaOeaaiabgUcaRmaalaaabaGae83Wdm3aa0baaSqaaiabdweafbqaaiabikdaYaaaaOqaaiabdQeakjabdUealbaacqGGPaqkaaa@5EB1@. Then,

SSA=JK∑i(Yi••−Y•••)2=JK∑i(αi+Si−S•)2.
 MathType@MTEF@5@5@+=feaafiart1ev1aaatCvAUfKttLearuWrP9MDH5MBPbIqV92AaeXatLxBI9gBaebbnrfifHhDYfgasaacH8akY=wiFfYdH8Gipec8Eeeu0xXdbba9frFj0=OqFfea0dXdd9vqai=hGuQ8kuc9pgc9s8qqaq=dirpe0xb9q8qiLsFr0=vr0=vr0dc8meaabaqaciaacaGaaeqabaqabeGadaaakeaacqWGtbWucqWGtbWudaWgaaWcbaGaemyqaeeabeaakiabg2da9iabdQeakjabdUealnaaqafabaGaeiikaGIaemywaK1aaSbaaSqaaiabdMgaPjabgkci3kabgkci3cqabaGccqGHsislaSqaaiabdMgaPbqab0GaeyyeIuoakiabdMfaznaaBaaaleaacqGHIaYTcqGHIaYTcqGHIaYTaeqaaOGaeiykaKYaaWbaaSqabeaacqaIYaGmaaGccqGH9aqpcqWGkbGscqWGlbWsdaaeqbqaaiabcIcaOGGaciab=f7aHnaaBaaaleaacqWGPbqAaeqaaOGaey4kaSIaem4uam1aaSbaaSqaaiabdMgaPbqabaGccqGHsislcqWGtbWudaWgaaWcbaGaeyOiGClabeaakiabcMcaPaWcbaGaemyAaKgabeqdcqGHris5aOWaaWbaaSqabeaacqaIYaGmaaGccqGGUaGlaaa@5BEC@

The null distribution of *SS*_*A *_is a scaled central chi-squared random variable with scaling factor σE2+KσP¯2
 MathType@MTEF@5@5@+=feaafiart1ev1aaatCvAUfKttLearuWrP9MDH5MBPbIqV92AaeXatLxBI9gBaebbnrfifHhDYfgasaacH8akY=wiFfYdH8Gipec8Eeeu0xXdbba9frFj0=OqFfea0dXdd9vqai=hGuQ8kuc9pgc9s8qqaq=dirpe0xb9q8qiLsFr0=vr0=vr0dc8meaabaqaciaacaGaaeqabaqabeGadaaakeaaiiGacqWFdpWCdaqhaaWcbaGaemyraueabaGaeGOmaidaaOGaey4kaSIaem4saSKae83Wdm3aa0baaSqaaiqbdcfaqzaaraaabaGaeGOmaidaaaaa@36D1@ so that

F1,2(J−1)=SSA/1SSP/2(J−1)
 MathType@MTEF@5@5@+=feaafiart1ev1aaatCvAUfKttLearuWrP9MDH5MBPbIqV92AaeXatLxBI9gBaebbnrfifHhDYfgasaacH8akY=wiFfYdH8Gipec8Eeeu0xXdbba9frFj0=OqFfea0dXdd9vqai=hGuQ8kuc9pgc9s8qqaq=dirpe0xb9q8qiLsFr0=vr0=vr0dc8meaabaqaciaacaGaaeqabaqabeGadaaakeaacqWGgbGrdaWgaaWcbaGaeGymaeJaeiilaWIaeGOmaiJaeiikaGIaemOsaOKaeyOeI0IaeGymaeJaeiykaKcabeaakiabg2da9maalaaabaGaem4uamLaem4uam1aaSbabSqaaiabdgeabbqabaGccqGGVaWlcqaIXaqmaeaacqWGtbWucqWGtbWudaWgaaWcbaGaemiuaafabeaakiabc+caViabikdaYiabcIcaOiabdQeakjabgkHiTiabigdaXiabcMcaPaaaaaa@4632@

has a central *F*-distribution with 1 numerator degree of freedom and 2(*J *- 1) denominator df when H_0_: *α*_*i *_≡ 0 is valid. Under the alternative hypothesis, the distribution of *SS*_*A *_is a weighted sum of non-central chi-squared random variables. The approximation to the alternative distribution of the *F*-test proposed here is that it is a non-central *F *with 1 numerator degree of freedom, 2(*J *- 1) denominator df, and non-centrality parameter (NCP) *δ*^2^, where

δ2=JK∑αi2(KσP¯2+σE2)=NK∑αi2T(KσP¯2+σE2).
 MathType@MTEF@5@5@+=feaafiart1ev1aaatCvAUfKttLearuWrP9MDH5MBPbIqV92AaeXatLxBI9gBaebbnrfifHhDYfgasaacH8akY=wiFfYdH8Gipec8Eeeu0xXdbba9frFj0=OqFfea0dXdd9vqai=hGuQ8kuc9pgc9s8qqaq=dirpe0xb9q8qiLsFr0=vr0=vr0dc8meaabaqaciaacaGaaeqabaqabeGadaaakeaaiiGacqWF0oazdaahaaWcbeqaaiabikdaYaaakiabg2da9maalaaabaGaemOsaOKaem4saS0aaabqaeaacqWFXoqydaqhaaWcbaGaemyAaKgabaGaeGOmaidaaaqabeqaniabggHiLdaakeaacqGGOaakcqWGlbWscqWFdpWCdaqhaaWcbaGafmiuaaLbaebaaeaacqaIYaGmaaGccqGHRaWkcqWFdpWCdaqhaaWcbaGaemyraueabaGaeGOmaidaaOGaeiykaKcaaiabg2da9maalaaabaGaemOta4Kaem4saS0aaabqaeaacqWFXoqydaqhaaWcbaGaemyAaKgabaGaeGOmaidaaaqabeqaniabggHiLdaakeaacqWGubavcqGGOaakcqWGlbWscqWFdpWCdaqhaaWcbaGafmiuaaLbaebaaeaacqaIYaGmaaGccqGHRaWkcqWFdpWCdaqhaaWcbaGaemyraueabaGaeGOmaidaaOGaeiykaKcaaiabc6caUaaa@5C3A@

As shown by Gronow [28], the inequality in variance does not affect the power approximation when *p *≤ 1.5 Since σE2=τ¯2T(m−1)
 MathType@MTEF@5@5@+=feaafiart1ev1aaatCvAUfKttLearuWrP9MDH5MBPbIqV92AaeXatLxBI9gBaebbnrfifHhDYfgasaacH8akY=wiFfYdH8Gipec8Eeeu0xXdbba9frFj0=OqFfea0dXdd9vqai=hGuQ8kuc9pgc9s8qqaq=dirpe0xb9q8qiLsFr0=vr0=vr0dc8meaabaqaciaacaGaaeqabaqabeGadaaakeaaiiGacqWFdpWCdaqhaaWcbaGaemyraueabaGaeGOmaidaaOGaeyypa0ZaaSaaaeaacuWFepaDgaqeamaaCaaaleqabaGaeGOmaidaaaGcbaGaemivaqfaaiabcIcaOiabd2gaTjabgkHiTiabigdaXiabcMcaPaaa@3AEC@, where 1 ≤ *m*,

δ2=JK∑αi2(KσP¯2+σE2)=NK∑αi2T[Kτ¯2T+τ¯2T(m−1)]=NK∑αi2[Kτ¯2+τ¯2(m−1)],
 MathType@MTEF@5@5@+=feaafiart1ev1aaatCvAUfKttLearuWrP9MDH5MBPbIqV92AaeXatLxBI9gBaebbnrfifHhDYfgasaacH8akY=wiFfYdH8Gipec8Eeeu0xXdbba9frFj0=OqFfea0dXdd9vqai=hGuQ8kuc9pgc9s8qqaq=dirpe0xb9q8qiLsFr0=vr0=vr0dc8meaabaqaciaacaGaaeqabaqabeGadaaakeaaiiGacqWF0oazdaahaaWcbeqaaiabikdaYaaakiabg2da9maalaaabaGaemOsaOKaem4saS0aaabqaeaacqWFXoqydaqhaaWcbaGaemyAaKgabaGaeGOmaidaaaqabeqaniabggHiLdaakeaacqGGOaakcqWGlbWscqWFdpWCdaqhaaWcbaGafmiuaaLbaebaaeaacqaIYaGmaaGccqGHRaWkcqWFdpWCdaqhaaWcbaGaemyraueabaGaeGOmaidaaOGaeiykaKcaaiabg2da9maalaaabaGaemOta4Kaem4saS0aaabqaeaacqWFXoqydaqhaaWcbaGaemyAaKgabaGaeGOmaidaaaqabeqaniabggHiLdaakeaacqWGubavcqGGBbWwcqWGlbWsdaWcaaqaaiqb=r8a0zaaraWaaWbaaSqabeaacqaIYaGmaaaakeaacqWGubavaaGaey4kaSYaaSaaaeaacuWFepaDgaqeamaaCaaaleqabaGaeGOmaidaaaGcbaGaemivaqfaaiabcIcaOiabd2gaTjabgkHiTiabigdaXiabcMcaPiabc2faDbaacqGH9aqpdaWcaaqaaiabd6eaojabdUealnaaqaeabaGae8xSde2aa0baaSqaaiabdMgaPbqaaiabikdaYaaaaeqabeqdcqGHris5aaGcbaGaei4waSLaem4saSKaf8hXdqNbaebadaahaaWcbeqaaiabikdaYaaakiabgUcaRiqb=r8a0zaaraWaaWbaaSqabeaacqaIYaGmaaGccqGGOaakcqWGTbqBcqGHsislcqaIXaqmcqGGPaqkcqGGDbqxaaGaeiilaWcaaa@7B4E@

which is not dependent upon *J*, assuming this model. This result is due to the fact that we assumed σP,i2=τi2T
 MathType@MTEF@5@5@+=feaafiart1ev1aaatCvAUfKttLearuWrP9MDH5MBPbIqV92AaeXatLxBI9gBaebbnrfifHhDYfgasaacH8akY=wiFfYdH8Gipec8Eeeu0xXdbba9frFj0=OqFfea0dXdd9vqai=hGuQ8kuc9pgc9s8qqaq=dirpe0xb9q8qiLsFr0=vr0=vr0dc8meaabaqaciaacaGaaeqabaqabeGadaaakeaaiiGacqWFdpWCdaqhaaWcbaGaemiuaaLaeiilaWIaemyAaKgabaGaeGOmaidaaOGaeyypa0ZaaSaaaeaacqWFepaDdaqhaaWcbaGaemyAaKgabaGaeGOmaidaaaGcbaGaemivaqfaaaaa@398E@, which is an assumption that each individual's variance contributes equally to the variance of the pool. The factor (*m *- 1) includes the cumulative effect of such sources of additional variability as experimental error, differential variability in processing of individuals, and other sources.

### Multiple regression analysis of approximate power

We calculated the approximate power of the experimental design under various values of parameters (Table 3). We then used OLS multiple regression analysis to identify the parameters that had the greatest impact on power, using SAS software [30]. For independent variables, we used all variables listed in Table 3, all two way interactions of these variables, and *K*^2^, the square of the number of replicates to incorporate the existence of an optimal number of replicates. We considered type I errors at 0.01, 0.001 and 0.0001 levels. It might be argued that researchers should use 0.0001 or less as a stringent significance level if the design is applied in a genome-wide association study. Since DNA pooling techniques are normally used as 1^st ^stage screening and for 1^st ^stage design, researchers may be more concerned with false negatives than false positives [9,31].

## Authors' contributions

FJ, SJF, NRM, and DG conceived of the study design. FJ performed all statistical analyses presented in the manuscript. CH wrote software to assist FJ in her analyses. FJ, SJF, and DG wrote the original manuscript and all revisions. All authors have read and approve the final manuscript.
